# Supplementary material for: Alcohol use recording in adults with depression in English primary care: a cross-sectional study
Source: BMJ Open. 2022 Jan 21;12(1):e055975. doi: 10.1136/bmjopen-2021-055975 (PMC8785169; doi:10.1136/bmjopen-2021-055975)
Supplement: Supplementary data [file bmjopen-2021-055975supp001.pdf]

Supplementary material

Figure S1: Cohort selection flowchart

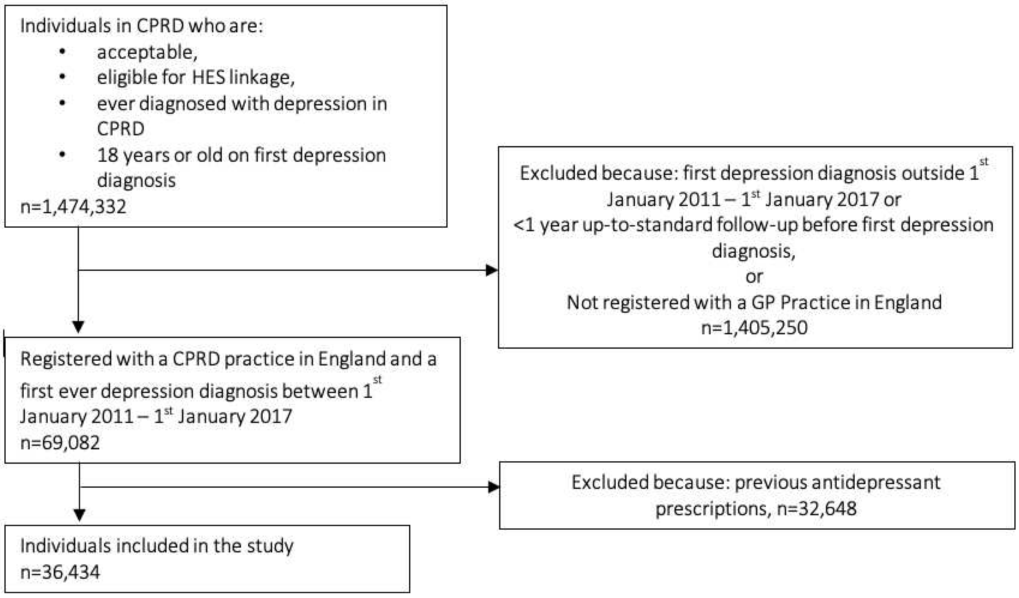

Abbreviations: CPRD – Clinical Practice Research Datalink; HES – Hospital Episode Statistics

Algorithms used to identify BMI and smoking status

Both smoking status and BMI were identified using an algorithm that utilised primary care records to identify the status recorded closest to the date of depression diagnosis. For BMI, Read codes for BMI category were not used because they are rarely recorded. Instead, BMI was calculated using height and weight measures recorded closest to the date of depression diagnosis. In the algorithm, records within -1 year to +1 month of the date of depression diagnosis were regarded as the best, +1 month to +1 year from the date of depression diagnosis as second best, the nearest prior to the year before the date of depression diagnosis as the third best, and within +1 year from the date of depression diagnosis as the worst. BMI was categorised as underweight (<18.5kg/m^2), normal (18.5 – <25.0 kg/m^2) or overweight (≥25.0 kg/m^2). Smoking status was classified as current smoker, ex-smoker, or non-smoker.

Table 1: Characteristics of individuals in the study and the odds of alcohol-use recording in the three months before or after depression diagnosis (data are N(%) unless otherwise specified)

	Alcohol use recording in primary care electronic health records		Age, sex, ethnicity, and deprivation adjusted odds ratio for alcohol use recording (95% CI)[b]
	Recorded [a]	Not recorded	
	N=6,012 (16.5%)	N=30,422 (83.5%)	
Sex			
Female	2,791 (14.2)	16,813 (85.8)	reference
Male	3,221 (19.1)	13,609 (80.9)	1.38 (1.29, 1.48)
Age at depression diagnosis (years)			
18-29	1,555 (13.3)	10,126 (86.7)	reference
30-39	1,044 (13.8)	6,507 (86.2)	1.05 (0.95, 1.17)
40-49	1,329 (18.5)	5,863 (81.5)	1.63 (1.47, 1.80)
50-59	943 (19.7)	3,854 (80.3)	1.81 (1.62, 2.02)
60-69	545 (23.1)	1,816 (76.9)	2.13 (1.86, 2.44)
≥70	596 (20.9)	2,256 (79.1)	1.85 (1.61, 2.11)
Ethnicity			
White	3,530 (17.4)	16,739 (82.6)	reference
South Asian	152 (17.8)	700 (82.2)	1.08 (0.90, 1.30)
Black	100 (16.8)	495 (83.2)	0.92 (0.74, 1.15)
Other	58 (15.6)	315 (84.5)	0.87 (0.65, 1.15)
Mixed	43 (16.5)	217 (83.5)	1.03 (0.74, 1.44)
Unknown	194 (18.7)	843 (81.3)	1.13 (0.96, 1.33)
Missing	*1,935 (14.8)*	*11,113 (85.17)*	
Geographical region			
South	3,623 (14.9)	20,632 (85.1)	reference
North	2,389 (19.6)	9,790 (80.4)	1.41 (1.31, 1.52)
IMD quintile			
1 – most deprived	1,123 (14.8)	6,479 (85.2)	reference
2	1,125 (15.2)	6,283 (84.8)	1.02 (0.91, 1.14)
3	1,241 (16.4)	6,324 (83.6)	1.15 (1.03, 1.28)
4	1,250 (17.2)	6,003 (82.8)	1.26 (1.12, 1.40)
5 – least deprived	1,269 (19.3)	5,316 (80.7)	1.56 (1.40, 1.75)
Missing	*4 (19.1)*	*17 (80.9)*	
Time (t) since GP registration (years)			
1<t≤2	587 (15.1)	3,313 (84.9)	reference
2<t≤3	410 (14.7)	2,374 (85.3)	0.95 (0.83, 1.10)
>3	3,015 (16.9)	24,735 (83.1)	1.11 (1.00, 1.23)
BMI Category			
Normal (18.5 – <25.0 kg/m^2)	2,037 (16.6)	10,217 (83.4)	reference
Underweight (<18.5kg/m^2)	176 (16.0)	922 (84.0)	0.95 (0.77, 1.17)
Overweight (≥25.0 kg/m^2)	3,221 (18.3)	14,428 (81.7)	0.98 (0.91, 1.06)
Missing	*578 (10.6)*	*4,855 (89.4)*	
Smoking Status			
Non-smoker	2,199 (14.4)	13,114 (85.6)	reference
Current smoker	1,911 (18.6)	8,342 (81.4)	1.34 (1.23, 1.46)
Ex-smoker	1,888 (17.8)	8,704 (82.2)	1.14 (1.05, 1.24)

Missing	*14 (5.1)*	*262 (94.9)*	
Comorbidities [c]			
Diabetes mellitus			
None	5,402 (15.6)	29,219 (84.4)	reference
Type 1	63 (31.2)	139 (68.8)	2.22 (1.53, 3.22)
Type 2	452 (33.9)	883 (66.1)	1.95 (1.67, 2.27)
Type unspecified	95 (34.4)	181 (65.6)	2.18 (1.59, 2.98)
Hypertension			
None	4,852 (15.3)	26,916 (84.7)	reference
Present	1,160 (24.9)	3,506 (75.1)	1.52 (1.37, 1.70)
Liver Disease			
None	5,908 (16.4)	30,046 (83.6)	reference
Yes	104 (21.7)	376 (78.3)	0.99 (0.75, 1.31)
Anxiety			
None	4,681 (16.6)	23,603 (83.4)	reference
Yes	1,331 (16.3)	6,819 (83.7)	0.98 (0.90, 1.07)
Substance Abuse			
None	5,951 (16.5)	30,195 (83.5)	reference
Yes	61 (21.2)	227 (78.8)	0.99 (0.69, 1.44)
Depression Management			
Antidepressant class			
None	744 (17.8)	3,448 (82.2)	reference
TCAs	139 (18.9)	596 (81.1)	1.13 (0.89, 1.44)
SSRIs	4,771 (16.1)	24,945 (83.9)	0.92 (0.82, 1.02)
Other	358 (20.0)	1,433 (80.0)	1.05 (0.88, 1.25)
Talking Therapies			
No	5,491 (16.4)	28,045 (83.6)	reference
Yes	521 (18.0)	2,377 (82.0)	1.20 (1.06, 1.36)

Abbreviations: TCAs – tricyclic antidepressants; SSRIs – selective serotonin uptake inhibitors
[a] The first instance of alcohol use recording in the three months before or after depression diagnosis
[b] Odds ratio adjusted for sociodemographic confounders (age, sex, ethnicity and deprivation)
