# Supplementary material for: Alcohol use recording in adults with depression in English primary care: a cross-sectional study
Source: BMJ Open. 2022 Jan 21;12(1):e055975. doi: 10.1136/bmjopen-2021-055975 (PMC8785169; doi:10.1136/bmjopen-2021-055975)
Supplement: Author's manuscript [file bmjopen-2021-055975.draft_revisions.pdf]

BMJ Open

BMJ Open is committed to open peer review. As part of this commitment we make the peer review history of every article we publish publicly available.

When an article is published we post the peer reviewers' comments and the authors' responses online. We also post the versions of the paper that were used during peer review. These are the versions that the peer review comments apply to.

The versions of the paper that follow are the versions that were submitted during the peer review process. They are not the versions of record or the final published versions. They should not be cited or distributed as the published version of this manuscript.

BMJ Open is an open access journal and the full, final, typeset and author-corrected version of record of the manuscript is available on our site with no access controls, subscription charges or pay-per-view fees (http://bmjopen.bmj.com).

If you have any questions on BMJ Open's open peer review process please email info.bmjopen@bmj.com

BMJ Open

Alcohol use recording in adults with depression in English primary care: a cross-sectional study

Journal:	*BMJ Open*
Manuscript ID	bmjopen-2021-055975
Article Type:	Original research
Date Submitted by the Author:	31-Jul-2021
Complete List of Authors:	Adesanya, Elizabeth; LSHTM, Department of Non-Communicable Disease Epidemiology<br>Cook, Sarah; LSHTM, Department of Non-Communicable Disease Epidemiology<br>Crellin, Elizabeth; The Health Foundation<br>Langan, Sinead; London School of Hygiene & Tropical Medicine, Department of Non-Communicable Disease Epidemiology<br>Mansfield, Kathryn; LSHTM, Department of Non-Communicable Disease Epidemiology<br>Smeeth, Liam; LSHTM, Department of Non-Communicable Disease Epidemiology<br>Herrett, Emily; LSHTM, Department of Non-Communicable Disease Epidemiology
Keywords:	EPIDEMIOLOGY, PRIMARY CARE, Depression & mood disorders < PSYCHIATRY, Substance misuse < PSYCHIATRY

SCHOLARONE™
Manuscripts

I, the Submitting Author has the right to grant and does grant on behalf of all authors of the Work (as defined in the below author licence), an exclusive licence and/or a non-exclusive licence for contributions from authors who are: i) UK Crown employees; ii) where BMJ has agreed a CC-BY licence shall apply, and/or iii) in accordance with the terms applicable for US Federal Government officers or employees acting as part of their official duties; on a worldwide, perpetual, irrevocable, royalty-free basis to BMJ Publishing Group Ltd ("BMJ") its licensees and where the relevant Journal is co-owned by BMJ to the co-owners of the Journal, to publish the Work in this journal and any other BMJ products and to exploit all rights, as set out in our licence.

The Submitting Author accepts and understands that any supply made under these terms is made by BMJ to the Submitting Author unless you are acting as an employee on behalf of your employer or a postgraduate student of an affiliated institution which is paying any applicable article publishing charge ("APC") for Open Access articles. Where the Submitting Author wishes to make the Work available on an Open Access basis (and intends to pay the relevant APC), the terms of reuse of such Open Access shall be governed by a Creative Commons licence – details of these licences and which Creative Commons licence will apply to this Work are set out in our licence referred to above.

Other than as permitted in any relevant BMJ Author's Self Archiving Policies, I confirm this Work has not been accepted for publication elsewhere, is not being considered for publication elsewhere and does not duplicate material already published. I confirm all authors consent to publication of this Work and authorise the granting of this licence.

For peer review only

Alcohol use recording in adults with depression in English primary care: a cross-sectional study

Authors: Elizabeth I. Adesanya,[1] Sarah Cook,[1,2] Elizabeth Crellin,[3] Sinéad M. Langan,[1] Kathryn E. Mansfield,[1] Liam Smeeth,[1] Emily Herrett[1]

Affiliations

[1] Department of Non-Communicable Disease Epidemiology, London School of Hygiene and Tropical Medicine, London, WC1E 7HT, United Kingdom

[2] National Heart and Lung Institute, Imperial College London, London, SW3 6LY, United Kingdom

[3] The Health Foundation, London, EC4Y 8AP, United Kingdom

Corresponding author

Elizabeth Adesanya, elizabeth.adesanya@lshtm.ac.uk

London School of Hygiene and Tropical Medicine, Keppel Street, London, WC1E 7HT, United Kingdom

Word count

Abstract: 273 words

Main text: 3576 words (excluding title page, abstract, tables, acknowledgements, contributions, and references)

Tables: 3

Figures: 1

Supplementary Figure: 1

For peer review only

ABSTRACT

Objectives

To investigate alcohol-use recording in people with newly diagnosed depression in English primary care, and individual characteristics associated with the recording of alcohol use.

Design

A population-based cross-sectional study.

Setting

Primary care data from English practices contributing to the UK Clinical Practice Research Datalink.

Participants

We included adults (18yrs+) diagnosed with depression between 1st January 2011 – 1st January 2017 without previous antidepressant use and at least one year of registration before diagnosis.

Primary and secondary outcome measures

We described the proportion of individuals with alcohol use records and recordings of the level of alcohol use at four timepoints (the date of depression diagnosis, three months before or after depression diagnosis, 12 months before or after depression diagnosis, and any point pre- or post-depression diagnosis). We used logistic regression to investigate individual characteristics associated with alcohol-use recording in the three months before or after depression diagnosis.

Results

For peer review only

We identified 36,424 adults with depression. 538 (2%) had alcohol use recorded in the three months before or after depression diagnosis using formal validated methods such as the Alcohol Use Disorders Identification Test and its abbreviated versions. At each timepoint, most individuals with alcohol use recorded were low risk drinkers. Alcohol-use recording in the three months before or after depression diagnosis was associated with male sex (OR=1.38, 95% CI: 1.29,1.48) and several other individual-level factors.

Conclusions

Our study shows low levels of alcohol-use recording in the three months before or after depression diagnosis. Levels of alcohol-use recording varied depending on individual characteristics. Incentivised recording of alcohol use will increase completeness, which will improve clinical management and reduce missed opportunities for care in people with depression.

For peer review only

ARTICLE SUMMARY

Strengths and limitations of this study

- Our study investigated to what extent alcohol use is recorded in the primary care health records of adults diagnosed with depression in England.
- We demonstrated that alcohol-use recording mainly takes place using informal methods, and the use of formal, validated tools, such as the AUDIT or FAST, is low (2% of people had alcohol use recorded in the three months before or after depression diagnosis).
- However, while our study suggests low levels of formal alcohol-use recording in primary care, it does not mean that alcohol use is not discussed with GPs in individuals with depression.

For peer review only

INTRODUCTION

Harmful alcohol use is a leading risk factor for premature death and disability.[1] In England, 22% of adults consume harmful levels of alcohol,[2] with alcohol-related harm costing the National Health Service (NHS) £3.5 billion annually.[3] Harmful alcohol use has been linked with the common mental health condition of depression.[4] In 2014, 20% of people aged 16 and over in the UK had symptoms of anxiety or depression.[5] The relationship between harmful alcohol use and depression is bidirectional – people with depression are more likely to drink harmfully, and, harmful levels of alcohol can act as a depressant, leading people who drink to excess experiencing symptoms of depression.[6,7] Furthermore, harmful alcohol use presents numerous challenges for depression management by affecting depression symptoms and, adherence and response to treatment.[8]

Screening for alcohol use allows early detection of harmful alcohol use and subsequent intervention before health issues become pronounced or irreversible.[9] UK National Institute for Health and Care Excellence (NICE) guidelines published in 2010 emphasise alcohol screening in primary care for subgroups at increased risk of alcohol abuse, including people with depression.[10] The Alcohol Use Disorders Identification Test (AUDIT) is a commonly used screening tool, and abbreviated versions have been developed for rapid screening in primary care and emergency departments (Fast Alcohol Screening Test [FAST], AUDIT for consumption [AUDIT C]).[11] Alcohol use is also recorded in primary care health records using other measures such as drinking status and level of alcohol consumption.[12,13]

Given the burden of harmful alcohol use and depression in the UK, and the damaging effects of harmful alcohol use in people with depression, it is crucial that we understand the extent to which alcohol use is recorded in people with depression. Previous work has

For peer review only - http://bmjopen.bmj.com/site/about/guidelines.xhtml

examined alcohol-use recording generally in UK primary care,[12-14] but this is the first study to investigate alcohol-use recording specifically in people with newly diagnosed depression.

For peer review only

For peer review only - http://bmjopen.bmj.com/site/about/guidelines.xhtml

METHODS

Study design and setting

We identified adults (18+years) with depression using prospectively collected primary care electronic health record data from the UK Clinical Practice Research Datalink (CPRD) GOLD database,[15] and linked Index of Multiple Deprivation (IMD) data.[16] The CPRD is an ongoing, nationwide primary care database of routinely collected and anonymised medical records.[15] We included all adults (≥18 years) in the CPRD GOLD database registered with a CPRD practice in England with a first depression diagnosis recorded between 1st January 2011 and 1st January 2017. We excluded individuals if they had less than one year of research standard registration prior to their depression diagnosis, or they had previously been prescribed antidepressants (to better capture newly diagnosed depression).

Factors associated with recording of alcohol use

We investigated a range of potential individual-level factors that could be associated with alcohol-use recording in primary care for people with depression: age at depression diagnosis, sex, ethnicity, deprivation, geographical region, smoking status, body mass index (BMI), the number of years after GP registration that depression was diagnosed (1-2 years, 2-3 years, >3 years), depression management with medication (antidepressant classes: tricyclic antidepressants [TCAs], selective serotonin uptake inhibitors [SSRIs], monoamine oxidase inhibitors [MAOIs], and other antidepressants) or without medication (talking therapies, e.g., cognitive behavioural therapy), and selected comorbidities related to alcohol use (liver disease, hypertension, diabetes mellitus, anxiety and substance abuse).

We defined deprivation using quintiles of the Index of Multiple Deprivation (IMD). We identified liver disease, hypertension and diabetes mellitus based on the presence of a

For peer review only - http://bmjopen.bmj.com/site/about/guidelines.xhtml

recorded (morbidity-coded) diagnosis in primary care prior to the date of the first depression diagnosis. We identified anxiety and substance abuse based on the presence of a (morbidity-coded) diagnosis up to five years prior to the date of the first depression diagnosis. We used existing code lists and algorithms to define BMI, ethnicity, and smoking status.[17,18] For BMI and smoking status, we used the status recorded closest to the date of the first depression diagnosis. We identified depression management with medication using primary care prescription data recorded after the first ever depression diagnosis. We identified talking therapies based on the presence of morbidity codes for psychological therapies (e.g., cognitive behavioural therapy, counselling). We have made all code lists used in this study available to download from online repositories.[19,20] Algorithms used to identify BMI and smoking status are described in further detail in the supplementary material.

Alcohol use recording

We identified recorded alcohol use using primary care morbidity coding (Read codes) and data captured in structured data-entry fields. Records of alcohol use included:

1. Codes indicating the use of AUDIT, AUDIT-C or FAST screening and their associated scores.
2. Codes indicating the diagnosis of alcohol use disorders (AUDs).
3. Codes quantifying alcohol use:
    a. Drinking status (current, non, ex).
    b. Drinking level (Read coded as light, moderate, heavy, or non).
    c. Units consumed per week (none, 1-14, 15-42, 43+).

For peer review only - http://bmjopen.bmj.com/site/about/guidelines.xhtml

We categorised alcohol use recording in terms of: (1) Type: whether it was formal (recorded using formal, validated methods, i.e., AUDIT, AUDIT-C or FAST), or informal (i.e., morbidity codes suggesting individuals were asked about drinking status or alcohol intake, or had an AUD diagnosis); and (2) Timing: of recorded alcohol use in relation to depression diagnosis at four time points (date of depression diagnosis; in the three months before or after depression diagnosis; in the 12 months before or after depression diagnosis; or at any time pre-or post-depression diagnosis).

Statistical analysis

We described the prevalence and level of recorded alcohol use in individuals diagnosed with depression according to whether it was: (1) formal or informal; and (2) timing in relation to date of depression diagnosis. Individuals whose alcohol use was recorded using formal, validated screening tools (e.g., AUDIT) had their level of alcohol use described according to the recorded screening tool scores. Individuals whose alcohol use was recorded using informal methods (e.g., drinking status or alcohol consumption) had their level of alcohol use based on the recorded information (e.g., units per week). Individuals in the study may have been recorded more than once at each timepoint and using more than one method of alcohol use recording. We used logistic regression to estimate odds ratios (adjusted for age, sex, ethnicity, and deprivation) for the association between the factors potentially associated with alcohol-use recording in the three months before or after depression diagnosis. Individuals with missing data on any of the variables considered were excluded from regression analyses. Data were analysed using Stata V.15 (StataCorp, Texas, USA).

Patient and public involvement

Patients or the public were not involved in the design or analysis of this study.

For peer review only

For peer review only - http://bmjopen.bmj.com/site/about/guidelines.xhtml

For peer review only

For peer review only - http://bmjopen.bmj.com/site/about/guidelines.xhtml

RESULTS

Study population

We identified 36,434 adults with a depression diagnosis recorded between 1st January 2011 and 1st January 2017 who were eligible for study inclusion **(Supplementary Figure 1)**. Fifty-four percent (n=19,604) of the study population were female, and the median age was 38 (IQR=27-51). There was an approximately even distribution of the study population across the deprivation quintiles.

Prevalence of alcohol use recording and level of alcohol use among those recorded

31,007 (85%) individuals had their alcohol use recorded at any point pre-or post-depression diagnosis. However, only 6,012 (17%) had their alcohol use recorded in the three months before or after their depression diagnosis **(Figure 1)**.

Formal alcohol use recording

Alcohol use recording using formal validated methods (AUDIT, AUDIT-C or FAST) was low; 23% (n=8,284) of the study population had their alcohol use recorded at any point pre- or post-depression diagnosis, and only 2% (n=538) had formal alcohol screening recorded in the three months before or after depression diagnosis **(Table 1)**. Most individuals who had their alcohol use recorded at each of the timepoints using AUDIT or FAST were identified as low risk drinkers **(Table 1)**. In the three months before or after depression diagnosis, 17% (63/370) of people who had their alcohol use recorded using AUDIT, and 17% (20/116) of those recorded using FAST, were identified as harmful drinkers (i.e., score of ≥8 on AUDIT tool, and ≥3 on FAST tool). In the three months before or after depression diagnosis, 18% (67/370) of those with their alcohol use recorded using AUDIT, and 19% recorded using FAST (22/116), had invalid scores recorded (i.e., score of >40 on AUDIT tool, and >16 on

For peer review only - http://bmjopen.bmj.com/site/about/guidelines.xhtml
For peer review only

FAST tool). Over 90% of individuals at each of the four timepoints who were screened with AUDIT-C did not have a score recorded by their GP.

Informal alcohol use recording

Sixteen percent (n=5,724) of the study population had their alcohol use recorded using informal methods (i.e., codes suggesting that individuals were asked about drinking status or alcohol intake, or had an AUD diagnosed) in the three months before or after depression diagnosis, and 83% (n=30,175) of individuals were recorded at any point pre- or post-depression diagnosis **(Table 2)**. Of the 51% (n=2,915) of individuals who had their weekly alcohol consumption recorded (a method of informal alcohol use recording) in the three months before or after depression diagnosis, 56% (n=1,621) were low risk drinkers, while 37% (n=1,087) were harmful drinkers (defined as consuming more than the recommended 14 units a week for men and women). Twenty-five percent (n=6,818) of individuals who had their weekly alcohol consumption recorded (in units per week) at any point pre-or post-depression diagnosis were harmful drinkers.

Factors associated with alcohol use recording

We saw evidence of increased odds of alcohol use recording using both formal validated methods (AUDIT, AUDIT-C and FAST) and informal methods (drinking level, alcohol consumption, drinking status) in the three months before or after depression diagnosis after adjusting for age, sex, ethnicity, and deprivation in: men (OR=1.38, 95% CI: 1.29, 1.48), people living in the North of England (OR=1.41, 95% CI: 1.31, 1.52) compared to those living in the South, current (OR=1.34, 95% CI: 1.23, 1.46) or ex-smokers (OR=1.14, 95% CI: 1.05, 1.24), individuals with their depression managed through talking therapies (OR=1.20, 95% CI: 1.06, 1.36) compared to those that go without, and people with hypertension (OR=1.52,

For peer review only - http://bmjopen.bmj.com/site/about/guidelines.xhtml

95% CI: 1.37, 1.70) or diabetes (Type 1 [OR=2.22, 95% CI:1.53,3.22]; Type 2 [OR=1.95, 95% CI: 1.67,2.27]; unspecified types [OR=2.18, 95% CI: 1.59, 2.98]) **(Table 3)**. There was also evidence of linear associations between alcohol-use recording and deprivation, and alcohol-use recording and time since GP registration. Individuals in the least deprived quintile of IMD were more likely to have their alcohol use recorded than the most deprived individuals (OR=1.56, 95% CI: 1.40, 1.75). Those registered at their GP practice for more than three years had 11% higher odds (OR=1.11, 95% CI: 1.00, 1.23) of alcohol use recording compared to those registered between 1-2 years **(Table 3)**. Age at depression diagnosis showed evidence of a strong non-linear ($p<0.001$) association with alcohol use recording. Individuals aged between 60-69 had more than twice the odds of alcohol use recording compared to those aged 18-29 (OR=2.13, 95% CI: 1.86,2.44).

For peer review only - http://bmjopen.bmj.com/site/about/guidelines.xhtml

Table 1: Number of individuals with alcohol use recorded using formal validated methods (AUDIT, AUDIT-C or FAST) at pre-specified timepoints in relation to depression diagnosis (data are N(%) unless otherwise specified)

	Timing of alcohol-use recording in relation to depression diagnosis N (%)			
	On date of depression diagnosis	+/- 3 months	+/- 12 months	Any point
Formal alcohol use recording (any) [a]	93 (0.3)	538 (1.5)	1,515 (4.2)	8,284 (22.7)
**AUDIT [b]**	**68 (73.1)**	**370 (68.8)**	**1,029 (67.9)**	**4,615 (55.7)**
Low risk (AUDIT score 0-7)	34 (50.0)	240 (64.9)	739 (71.8)	3,193 (69.2)
Hazardous (AUDIT score 8-15)	11 (16.2)	43 (11.6)	95 (9.2)	318 (6.9)
High risk (AUDIT score 16-19)	*	7 (1.9)	10 (1.0)	27 (0.6)
Possible dependence (AUDIT score 20-40)	*	13 (3.5)	18 (1.8)	37 (0.8)
Invalid score value recorded (AUDIT score >40)	17 (25.0)	67 (18.1)	167 (16.2)	1,040 (22.5)
**AUDIT-C [b]**	**22 (23.7)**	**139 (25.8)**	**395 (26.1)**	**2,886 (34.8)**
Low risk (AUDIT-C score 0-4)	0 (0.0)	10 (7.2)	22 (5.6)	79 (2.7)
Hazardous (AUDIT-C score 5-7)	0 (0.0)	*	*	19 (0.7)
High risk (AUDIT-C score 8-10)	0 (0.0)	0 (0.0)	*	6 (0.2)
Possible dependence (AUDIT-C score 11-12)	0 (0.0)	0 (0.0)	0 (0.0)	0 (0.0)
AUDIT-C score not recorded	22 (100.00)	128 (92.1)	366 (92.7)	2,782 (96.4)
**FAST [b]**	**16 (17.2)**	**116 (21.6)**	**368 (24.3)**	**2,849 (34.4)**
Low risk (FAST score 0-2)	11 (68.8)	74 (63.8)	242 (65.8)	1,633 (57.3)
Hazardous (FAST score 3-16)	*	20 (17.2)	48 (13.0)	336 (11.8)
Invalid score value recorded (FAST score >16)	*	22 (19.0)	78 (21.2)	880 (30.9)

For peer review only

Abbreviations: +/- 3 months – alcohol use recorded in the 3 months before or after depression diagnosis; +/- 12 months – alcohol use recorded in the year before or after depression diagnosis; any point – alcohol use recorded at any point pre- or post-depression diagnosis

[a] Percentage calculated out of the total number of individuals in the cohort (36,434)

[b] Percentage calculated out of the total number of individuals with alcohol use recorded using formal validated methods – individuals may have more than one type of formal recording, so numbers add to >100%; risk scores represent the proportion at risk identified using that particular test

[*] Cell counts less than 5 suppressed to preserve individuals' confidentiality.

For peer review only

Table 2: Number of individuals with alcohol use recorded using informal methods (i.e., alcohol status, level of alcohol use) at pre-specified timepoints in relation to depression diagnosis (data are N(%) unless otherwise specified)

	Timing of alcohol-use recording in relation to depression diagnosis N (%)			
	Date of depression diagnosis	+/- 3 months	+/- 12 months	Any point
Informal alcohol use recording (any) [a]	2,039 (5.6)	5,724 (15.7)	11,361 (31.2)	30,175 (82.8)
**Recording of current alcohol status [b]**	**901 (44.2)**	**3,025 (52.9)**	**6,643 (58.5)**	**24,195 (80.2)**
Non	313 (34.7)	947 (31.3)	2,081 (31.3)	8,145 (33.7)
Current	496 (55.1)	1,777 (58.7)	3,907 (58.8)	14,198 (58.7)
Ex	92 (10.2)	301 (10.0)	655 (9.9)	1,852 (7.7)
**Recording of codes indicating level of alcohol use [b]**	**855 (41.9)**	**2,893 (50.5)**	**6,406 (56.4)**	**24,377 (80.8)**
None	405 (47.4)	1,239 (42.8)	2,675 (41.8)	9,370 (38.4)
Light	304 (35.6)	1,260 (43.6)	2,956 (46.1)	11,383 (46.7)
Moderate	36 (4.2)	123 (4.3)	282 (4.4)	2,106 (8.6)
Heavy	110 (12.9)	271 (9.4)	493 (7.7)	1,518 (6.2)
**Recording of units of alcohol consumed per week [b]**	**1,090 (53.5)**	**2,915 (50.9)**	**6,390 (56.2)**	**27,377 (90.7)**
None	78 (7.2)	207 (7.1)	474 (7.4)	3,744 (13.7)
1-14	499 (45.8)	1,621 (55.6)	3,865 (60.5)	16,815 (61.4)
15-42	322 (29.5)	745 (25.6)	1,498 (23.4)	5,477 (20.0)
43+	191 (17.5)	342 (11.7)	553 (8.7)	1,341 (4.9)

For peer review only

For peer review only - http://bmjopen.bmj.com/site/about/guidelines.xhtml

**Recording of codes relating to alcohol use disorders [b]**	**39 (1.9)**	**117 (2.0)**	**213 (1.9)**	**584 (1.9)**

Abbreviations: +/- 3 months – alcohol use recorded in the 3 months before or after depression diagnosis; +/- 12 months – alcohol use recorded in the year before or after depression diagnosis; any point – alcohol use recorded at any point pre- or post-depression diagnosis

[a] Percentage calculated out of the total number of individuals in the cohort (36,434)

[b] Percentage calculated out of the total number of individuals with alcohol use recorded using informal methods – individuals may have more than one type of informal recording, so numbers add to >100%; percentages are out of the number of people with alcohol use recording using that specific method

For peer review only

For peer review only - http://bmjopen.bmj.com/site/about/guidelines.xhtml

Table 3: Characteristics of individuals in the study and the odds of alcohol-use recording in the three months before or after depression diagnosis (data are N(%) unless otherwise specified)

	Alcohol use recording in primary care electronic health records		Age, sex, ethnicity, and deprivation adjusted odds ratio for alcohol use recording (95% CI)[b]
	Recorded [a]	Not recorded	
	N=6,012 (16.5%)	N=30,422 (83.5%)	
**Sex**			
Female	2,791 (14.2)	16,813 (85.8)	reference
Male	3,221 (19.1)	13,609 (80.9)	1.38 (1.29, 1.48)
**Age at depression diagnosis (years)**			
18-29	1,555 (13.3)	10,126 (86.7)	reference
30-39	1,044 (13.8)	6,507 (86.2)	1.05 (0.95, 1.17)
40-49	1,329 (18.5)	5,863 (81.5)	1.63 (1.47, 1.80)
50-59	943 (19.7)	3,854 (80.3)	1.81 (1.62, 2.02)
60-69	545 (23.1)	1,816 (76.9)	2.13 (1.86, 2.44)
≥70	596 (20.9)	2,256 (79.1)	1.85 (1.61, 2.11)
**Ethnicity**			
White	3,530 (17.4)	16,739 (82.6)	reference
South Asian	152 (17.8)	700 (82.2)	1.08 (0.90, 1.30)
Black	100 (16.8)	495 (83.2)	0.92 (0.74, 1.15)
Other	58 (15.6)	315 (84.5)	0.87 (0.65, 1.15)
Mixed	43 (16.5)	217 (83.5)	1.03 (0.74, 1.44)
Unknown	194 (18.7)	843 (81.3)	1.13 (0.96, 1.33)
Missing	*1,935 (14.8)*	*11,113 (85.17)*	
**Geographical region**			
South	3,623 (14.9)	20,632 (85.1)	reference
North	2,389 (19.6)	9,790 (80.4)	1.41 (1.31, 1.52)
**IMD quintile**			

For peer review only

1 – most deprived	1,123 (14.8)	6,479 (85.2)	reference
2	1,125 (15.2)	6,283 (84.8)	1.02 (0.91, 1.14)
3	1,241 (16.4)	6,324 (83.6)	1.15 (1.03, 1.28)
4	1,250 (17.2)	6,003 (82.8)	1.26 (1.12, 1.40)
5 – least deprived	1,269 (19.3)	5,316 (80.7)	1.56 (1.40, 1.75)
Missing	*4 (19.1)*	*17 (80.9)*	
**Time (t) since GP registration (years)**			
1<t≤2	587 (15.1)	3,313 (84.9)	reference
2<t≤3	410 (14.7)	2,374 (85.3)	0.95 (0.83, 1.10)
>3	3,015 (16.9)	24,735 (83.1)	1.11 (1.00, 1.23)
**BMI Category**			
Normal (18.5 – <25.0 kg/m^2)	2,037 (16.6)	10,217 (83.4)	reference
Underweight (<18.5kg/m^2)	176 (16.0)	922 (84.0)	0.95 (0.77, 1.17)
Overweight (≥25.0 kg/m^2)	3,221 (18.3)	14,428 (81.7)	0.98 (0.91, 1.06)
Missing	*578 (10.6)*	*4,855 (89.4)*	
**Smoking Status**			
Non-smoker	2,199 (14.4)	13,114 (85.6)	1.0
Current smoker	1,911 (18.6)	8,342 (81.4)	1.34 (1.23, 1.46)
Ex-smoker	1,888 (17.8)	8,704 (82.2)	1.14 (1.05, 1.24)
Missing	*14 (5.1)*	*262 (94.9)*	
**Comorbidities** [c]			
Diabetes mellitus			
None	5,402 (15.6)	29,219 (84.4)	1.0
Type 1	63 (31.2)	139 (68.8)	2.22 (1.53, 3.22)
Type 2	452 (33.9)	883 (66.1)	1.95 (1.67, 2.27)
Type unspecified	95 (34.4)	181 (65.6)	2.18 (1.59, 2.98)
Hypertension			
None	4,852 (15.3)	26,916 (84.7)	1.0
Present	1,160 (24.9)	3,506 (75.1)	1.52 (1.37, 1.70)
Liver Disease			
None	5,908 (16.4)	30,046 (83.6)	1.0
Yes	104 (21.7)	376 (78.3)	0.99 (0.75, 1.31)
Anxiety			
None	4,681 (16.6)	23,603 (83.4)	1.0
Yes	1,331 (16.3)	6,819 (83.7)	0.98 (0.90, 1.07)
Substance Abuse			
None	5,951 (16.5)	30,195 (83.5)	1.0

For peer review only - http://bmjopen.bmj.com/site/about/guidelines.xhtml

For peer review only

Yes	61 (21.2)	227 (78.8)	0.99 (0.69, 1.44)
**Depression Management**			
Antidepressant class			
None	744 (17.8)	3,448 (82.2)	1.0
TCAs	139 (18.9)	596 (81.1)	1.13 (0.89, 1.44)
SSRIs	4,771 (16.1)	24,945 (83.9)	0.92 (0.82, 1.02)
Other	358 (20.0)	1,433 (80.0)	1.05 (0.88, 1.25)
Talking Therapies			
No	5,491 (16.4)	28,045 (83.6)	1.0
Yes	521 (18.0)	2,377 (82.0)	1.20 (1.06, 1.36)

Abbreviations: TCAs – tricyclic antidepressants; SSRIs – selective serotonin uptake inhibitors

[a] The first instance of alcohol use recording in the three months before or after depression diagnosis

[b] Odds ratio adjusted for sociodemographic confounders (age, sex, ethnicity and deprivation)

For peer review only

DISCUSSION

Summary

We found that levels of alcohol-use recording in people with depression were high: 85% of individuals had their alcohol use recorded using either formal or informal methods at any point pre-or post-depression diagnosis. However, only 17% of individuals had their alcohol use recorded in the three months before or after depression diagnosis, with only 2% of these individuals recorded using formal validated tools (AUDIT, AUDIT-C or FAST).

Just over one-third (37%) of individuals who had their weekly alcohol consumption recorded in the three months before or after depression diagnosis were harmful drinkers (defined as consuming more than the recommended 14 units a week for men and women), whereas 25% of individuals who had their weekly alcohol consumption recorded at any point pre- or post-depression diagnosis were harmful drinkers. After adjusting for age, sex, ethnicity, and deprivation, we found that alcohol-use recording in the three months before or after depression diagnosis was more likely for: men, people aged 60-69, people living in the North of England, current or ex-smokers, people with diabetes or hypertension, people in the least deprived areas, people registered at their GP practice for more than three years before their depression diagnosis, and people managing their depression using talking therapies.

Comparison with existing literature

Compared to a 2019 study, which showed that 52% of people in UK primary care had a record of alcohol use,[12] our study suggests that alcohol use recording in people with depression was higher, with 85% of individuals having their alcohol use recorded at any point pre-or post-depression diagnosis.

For peer review only - http://bmjopen.bmj.com/site/about/guidelines.xhtml

According to data from the 2018 Health Survey for England (HSE), 22% of all adults in England consumed more than the recommended 14 units a week.[2] This figure is comparable to the 25% of people with depression in this study who had their alcohol consumption recorded at any point pre- or post-depression diagnosis and were found to drink harmfully and consume more than the recommended weekly intake. However, the proportion of harmful drinkers identified by the HSE is lower than the proportion of individuals with depression in this study who had their alcohol consumption recorded in the three months before or after depression diagnosis and were found to be harmful drinkers (37%).

Both figures of harmful weekly alcohol consumption identified in this study (25% at any point pre- or post-depression diagnosis and 37% in the three months before or after depression diagnosis) are dissimilar to studies conducted in: 1) Singapore, where 19% of people with depression were harmful drinkers;[21] and 2) South Korea, where 51% of people with depression were harmful drinkers.[22] GP recording practices, as well as cultural and governmental variations, are possible explanations of this difference in results. Lower levels of harmful drinking in Singapore may be explained by strict alcohol laws; alcohol cannot be sold or consumed in public from 10:30pm to 7:00am.[21] Similarly, the prominent drinking culture in South Korea may contribute to the increased prevalence.[23]

Our study found that people with depression who were men, ex/current smokers, aged 60-69, diagnosed with diabetes or hypertension, from areas with the lowest level of deprivation, or who lived in the North of England were more likely to have their alcohol use recorded compared to other people with depression. Other UK primary care studies of alcohol-use recording have also demonstrated increased rates of alcohol-use recording in people with diabetes and hypertension, and those aged 60 to 69 years.[12-14] There is also evidence to suggest that some of the factors we identified as being linked to alcohol-use recording are

For peer review only - http://bmjopen.bmj.com/site/about/guidelines.xhtml
For peer review only

linked to harmful alcohol use. For example, smokers and ex-smokers have been shown to be more likely to consume harmful amounts of alcohol when compared to non-smokers.[24] Data from the 2018 HSE also showed that men were 15% more likely to drink harmfully compared to women, and the highest proportion of heavier drinkers was found in the North of England.[2] This evidence from HSE taken alongside our results may suggest that GPs are more likely to record alcohol use in those perceived to be at higher risk even amongst those with depression.

However, some characteristics that have been identified as being associated with increased levels of alcohol-use recording in our study contrast with finding from other UK studies. Other studies have shown that women are more likely to have their alcohol use recorded compared to men,[13] and those in the most deprived regions are more likely to be have recorded alcohol use than those in the least deprived areas.[12,13] However, the increased odds of alcohol use recording in the least deprived areas seen in this study may be explained by GP practices in more deprived areas possibly having fewer resources to deliver timely and formal alcohol-use recording.

Strengths and limitations

The CPRD GOLD dataset is broadly representative of the UK population with regards to age and sex,[15] and consequently our results can be broadly generalised to the rest of the UK population. Our study was able to use complete data on key variables such as age, sex, deprivation and comorbid conditions. Importantly this study captured levels of alcohol-use recording in a real life setting in UK primary care, and therefore highlights potential missed opportunities for care in people with depression.

For peer review only

An important limitation of this study is that individuals without alcohol use recorded may have discussed alcohol with their GP, but this was not documented leading to underestimation of alcohol-use screening based on recording as a proxy marker. Lack of documentation may be more likely where low levels of drinking were reported. While we cannot directly extrapolate our findings to discussion of alcohol use within GP consultations, our findings related to recording are important given documentation of alcohol use is crucial for informing future management and monitoring change in drinking.

Similarly, although our analysis found an association between various factors and alcohol-use recording, GPs may record alcohol use in individuals who they believe are at higher risk of harmful alcohol use for reasons that are not in their coded medical record. GPs may also base their decisions to ask about, and record, alcohol use on the previous alcohol level recorded. For instance, people who had previously had high levels of alcohol use recorded may be more likely to have their alcohol use recorded when they are diagnosed with depression. Equally the decision to record alcohol use if discussed in the consultation may also be related to the level of alcohol use (GPs may be more likely to document harmful drinking) which could influence the estimates of harmful drinking among those with depression found here.

Another limitation is the potential imprecision of alcohol consumption data recorded in primary care. A number of factors related to recording mean that our estimates may be an unreliable estimate of true alcohol intake. Firstly, we noted that some people with alcohol use recorded using formal methods either did not have a score for the test recorded (particularly frequent in those with alcohol use recorded using AUDIT-C), or the recorded score was outside the total possible value for the test. It is unclear how such missing data would affect our findings. Secondly, it is unclear whether individuals with recorded alcohol

For peer review only

use were asked about recent consumption (how much they actually consumed in the past week), or typical consumption (how much they typically consume in a week). The potential ambiguity in the consumption data implies differences in coding practices between individual GPs and between different GP practices. It also suggests that consumption records need to be used cautiously for research as they may not necessarily be capturing the same thing for each individual. And finally, individuals may be less truthful about their alcohol consumption if asked face-to-face when compared to a self-completed questionnaire (potentially underestimating harmful-drinking levels).

Data on variables such as substance abuse and depression management without medication are likely to be poorly captured within the CPRD. Restricting entry into the study to individuals without previous antidepressant prescriptions allowed us to more cleanly capture people with newly diagnosed depression, but it also reduced sensitivity and we may have missed individuals that had depression who had been prescribed antidepressants before depression diagnosis was coded.

Implication for research and/or practice

The results of this study offer an insight into alcohol-use recording for people with depression in English primary care. Our results suggest that NICE guidelines[10] have not been closely followed in primary care and GPs are not recording alcohol use in people with depression as effectively as they could be. If the low recording of codes relating to alcohol consumption, we saw represents genuine levels of alcohol-use screening in primary care, it suggests that alcohol use may not have been discussed and addressed by the GP, a potential missed opportunity for care in people with depression (who are at high risk of harmful alcohol use) that may lead to poorer outcomes. However, it is encouraging that

For peer review only - http://bmjopen.bmj.com/site/about/guidelines.xhtml

some alcohol use recording is taking place and that people with characteristics associated with harmful drinking are targeted.

The UK government recently introduced an, 'Alcohol related risk reduction scheme' that requires GPs to identify newly registered individuals aged 16 or over who are drinking harmful levels of alcohol,[25] however, there is no emphasis on depression. There is currently no financial incentive for recording alcohol use in people with depression through the Quality and Outcomes Framework (QOF),[26] even though in 2019, a NICE QOF indicator was suggested to record the percentage of individuals diagnosed with depression in the last year who had been screened for hazardous drinking in the three months before or after their depression diagnosis.[27] Additionally, the small financial reward offered to practices in England for screening newly registered adults for harmful alcohol use as part of Clinical Directed Enhanced Services (DES) ceased in 2015.[28,29] Evidence shows that the introduction of financial incentives leads to an increase in the numbers of people in primary care who have their alcohol use recorded.[30]

The incentivisation of alcohol use recording may help to increase numbers of people recorded, which is crucial for future patient management and will improve holistic care for individuals with depression. Adding alcohol-use recording as a QOF indicator or rewarding services for recording of alcohol use is likely to improve clinical practice and patient outcomes.

For peer review only - http://bmjopen.bmj.com/site/about/guidelines.xhtml

DECLARATIONS AND ACKNOWLEDGEMENTS

Contributions

EH, SC and KEM designed the study. EC and EH extracted the data. EIA performed the analysis and wrote the first draft of the manuscript. All authors (EIA, SC, EC, SML, KEM, LS and EH) reviewed and approved the final manuscript.

Acknowledgements

This study is based in part on data from the Clinical Practice Research Datalink obtained under licence from the UK Medicines and Healthcare products Regulatory Agency. The data is provided by patients and collected by the NHS as part of their care and support. The interpretation and conclusions contained in this study are those of the authors alone. The study was approved by the CPRD's Independent Scientific Advisory Committee (Protocol number: 19_077).

Funding

EH is funded by a post-doctoral fellowship from the NIHR (Reference: PDF-2016-09-029). SML is funded by a Wellcome Trust Senior Clinical Fellowship in Science (Reference: 205039/Z/16/Z). For the purpose of Open Access, the author has applied a CC BY public copyright licence to any Author Accepted Manuscript (AAM) version arising from this submission.

Ethical approval

The study was approved by the London School of Hygiene and Tropical Medicine Research Ethics Committee and by the Clinical Practice Research Datalink Independent Scientific Advisory Committee (ISAC Protocol Number: 19_077).

For peer review only - http://bmjopen.bmj.com/site/about/guidelines.xhtml
For peer review only

Competing interests

None declared

Data statement

Data were obtained from a third party and are not publicly available.

For peer review only

For peer review only - http://bmjopen.bmj.com/site/about/guidelines.xhtml

REFERENCES

1. World Health Organisation (WHO). *Alcohol*. Available from: https://www.who.int/health-topics/alcohol#tab=overview [Accessed 29th January 2020].

2. NHS Digital. *Health Survey for England (HSE) 2018*. Available from: https://digital.nhs.uk/data-and-information/publications/statistical/health-survey-for-england/2018 [Accessed 20th November 2020].

3. Institute of Alcohol Studies (IAS). *Estimates of the cost of alcohol*. Available from: http://www.ias.org.uk/Alcohol-knowledge-centre/Economic-impacts/Factsheets/Estimates-of-the-cost-of-alcohol.aspx [Accessed 20th November 2020].

4. Public Health England (PHE). *Health Matters: Harmful Drinking and Alcohol Dependence*. Available from: https://www.gov.uk/government/publications/health-matters-harmful-drinking-and-alcohol-dependence/health-matters-harmful-drinking-and-alcoholdependence#the-scale-of-the-problem [Accessed 29th January 2020].

5. Mental Health Foundation. *Fundamental Facts about Mental Health 2016*. Available from: https://www.mentalhealth.org.uk/file/2518/download?token=RU6lzcSK [Accessed 29th January 2020].

6. Boden JM, Fergusson DM. Alcohol and depression. *Addiction*. 2011;106(5):906-14. doi: 10.1111/j.1360-0443.2010.03351.x.

7. Ramsey SE, Engler PA, Stein MD. Alcohol Use Among Depressed Patients: The Need for Assessment and Intervention. *Prof Psychol Res Pr*. 2005;36(2):203-207. doi: 10.1037/0735-7028.36.2.203.

8. Hjorthøj C, Østergaard ML, Benros ME, et al. Association between alcohol and substance use disorders and all-cause and cause-specific mortality in schizophrenia, bipolar disorder, and unipolar depression: a nationwide, prospective, register-based study. *Lancet Psychiatry*. 2015;2(9):801–8. doi: 10.1016/S2215-0366(15)00207-2.

9. World Health Organisation (WHO). *Screening and brief intervention for alcohol problems in primary health care*. Available from: https://www.who.int/substance_abuse/activities/sbi/en/ [Accessed 29th January 2020].

10. National Institute for Health and Care Excellence (NICE). *Alcohol-use disorders: prevention.* Available from:

For peer review only - http://bmjopen.bmj.com/site/about/guidelines.xhtml

https://www.nice.org.uk/guidance/ph24/resources/alcoholuse-disorders-prevention-pdf-1996237007557 [Accessed 29th January 2020].

11. Reinert DF, Allen JP. The Alcohol Use Disorders Identification Test: An Update of Research Findings. *Alcohol Clin Exp Res*. 2007;31(2):185–99. doi: 10.1111/j.1530-0277.2006.00295.x.

12. Mansfield K, Crellin E, Denholm R, et al. Completeness and validity of alcohol recording in general practice within the UK: a cross-sectional study. *BMJ Open*. 2019;26;9(11):e031537. doi: 10.1136/bmjopen-2019-031537.

13. Khadjesari Z, Marston L, Petersen I, et al. Alcohol consumption screening of newly-registered patients in primary care: a cross-sectional analysis. *Br J Gen Pract*. 2013;63(615):706–12. doi: 10.3399/bjgp13X673720

14. Cheeta S, Drummond C, Oyefeso A, et al. Low identification of alcohol use disorders in general practice in England. *Addiction*. 2008;103(5):766-73. doi: 10.1111/j.1360-0443.2008.02198.x.

15. Herrett E, Gallagher AM, Bhaskaran K, et al. Data Resource Profile: Clinical Practice Research Datalink (CPRD). *Int J Epidemiol*. 2015;44(3):827–36. doi: 10.1093/ije/dyv098.

16. Department for Communities and Local Government. *The English Index of Multiple Deprivation (IMD) 2015 – Guidance*. Available from: https://assets.publishing.service.gov.uk/government/uploads/system/uploads/attachment_data/file/464430/English_Index_of_Multiple_Deprivation_2015_-_Guidance.pdf [Accessed 29th January 2020].

17. Mathur R, Bhaskaran K, Chaturvedi N, et al. Completeness and usability of ethnicity data in UK-based primary care and hospital databases. *J Public Health (Oxf)*. 2014;36(4):684-692. doi: 10.1093/pubmed/fdt116.

18. Bhaskaran K, Forbes HJ, Douglas I, et al. Representativeness and optimal use of body mass index (BMI) in the UK Clinical Practice Research Datalink (CPRD). *BMJ Open*. 2013;3: e003389. doi: 10.1136/bmjopen-2013-003389.

19. Adesanya E, Cook S, Mansfield KE, et al. Code lists for "Alcohol use recording in people with depression in English primary care". LSHTM Data Compass. London, United Kingdom: London School of Hygiene & Tropical Medicine; . Available from: https://doi.org/10.17037/DATA.00002183.

For peer review only

20. Herrett E, Cook S, Mansfield KE, et al. Code lists for "Completeness and validity of alcohol recording in general practice within the UK: a cross-sectional study". LSHTM Data Compass. London, United Kingdom: London School of Hygiene & Tropical Medicine; . Available from: https://doi.org/10.17037/data.00001071.

21. Subramaniam M, Mahesh MV, Peh CX, et al. Hazardous alcohol use among patients with schizophrenia and depression. *Alcohol*. 2017;65:63-69. doi: 10.1016/j.alcohol.2017.07.008.

22. Park SC, Lee SK, Oh HS, et al. Hazardous drinking-related characteristics of depressive disorders in Korea: The CRESCEND study. *J Korean Med Sci*. 2014;30(1):74-81. doi: 10.3346/jkms.2015.30.1.74.

23. Ryu SY, Crespi CM, Maxwell AE. Drinking patterns among Korean adults: Results of the 2009 Korean community health survey. *J Prev Med Public Health*. 2013;46(4):183-191. doi: 10.3961/jpmph.2013.46.4.183.

24. Chiolero A, Wietlisbach V, Ruffieux C, et al. Clustering of risk behaviours with cigarette consumption: A population-based study. *Prev Med*. 2006;42(5):348-353. doi: https://doi.org/10.1016/j.ypmed.2006.01.011.

25. NHS Digital. *Alcohol related risk reduction scheme*. Available from: https://digital.nhs.uk/services/general-practice-gp-collections/service-information/alcohol-related-risk-reduction-scheme-2017-18#top [Accessed 29th January 2020].

26. NHS Employers. *Quality and Outcomes Framework: Guidance for GMS contract 2019/20*. Available from: https://www.england.nhs.uk/wp-content/uploads/2019/05/gms-contract-qof-guidance-april-2019.pdf [Accessed 29th January 2020].

27. National Institute for Health and Care Excellence (NICE). *Quality and Outcomes Framework indicator.* Available from: https://www.nice.org.uk/standards-and-indicators/qofindicators/the-percentage-of-patients-with-a-new-diagnosis-of-depression-or-anxiety-in-the-preceding-12-months-who-have-been-screened-for-unsafe-drinking-using-the-fast-or-audit-c-tool-in-the-3-months-before-or-after-their-diagnosis-being-recorded [Accessed 29th January 2020].

28. NHS Employers. *Clinical directed enhanced services (DES) guidance for GMS contract 2008/09*. Available from:

For peer review only - http://bmjopen.bmj.com/site/about/guidelines.xhtml

https://www.lmc.org.uk/visageimages/guidance/2008/clinicaldesguidance.pdf [Accessed 29th January 2020].

29. Croner-i. *Enhanced Services: In-depth*. Available from: https://app.croneri.co.uk/topics/enhanced-services/indepth [Accessed 29th January 2020]

30. Hamilton FL, Laverty AA, Gluvajic D, et al. Effect of financial incentives on delivery of alcohol screening and brief intervention (ASBI) in primary care: longitudinal study. *J Public Health (Oxf)*. 2014;36(3):450-9. doi: 10.1093/pubmed/fdt121.

For peer review only

For peer review only - http://bmjopen.bmj.com/site/about/guidelines.xhtml

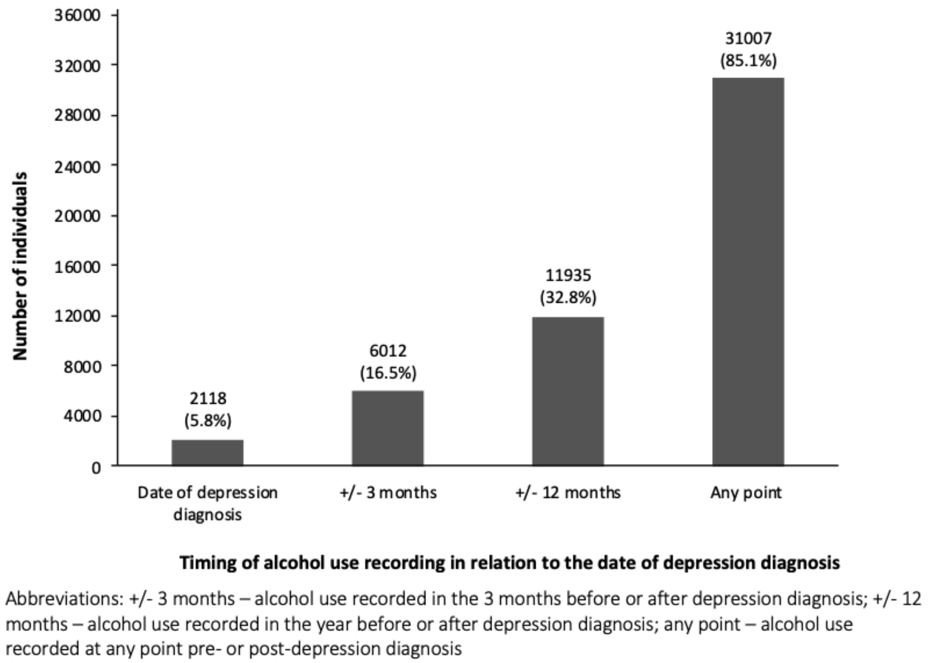

Figure 1: Number (%) of individuals with alcohol use recorded at times in relation to the date of their depression diagnosis.

303x209mm (72 x 72 DPI)

Supplementary material

Figure S1: Cohort selection flowchart

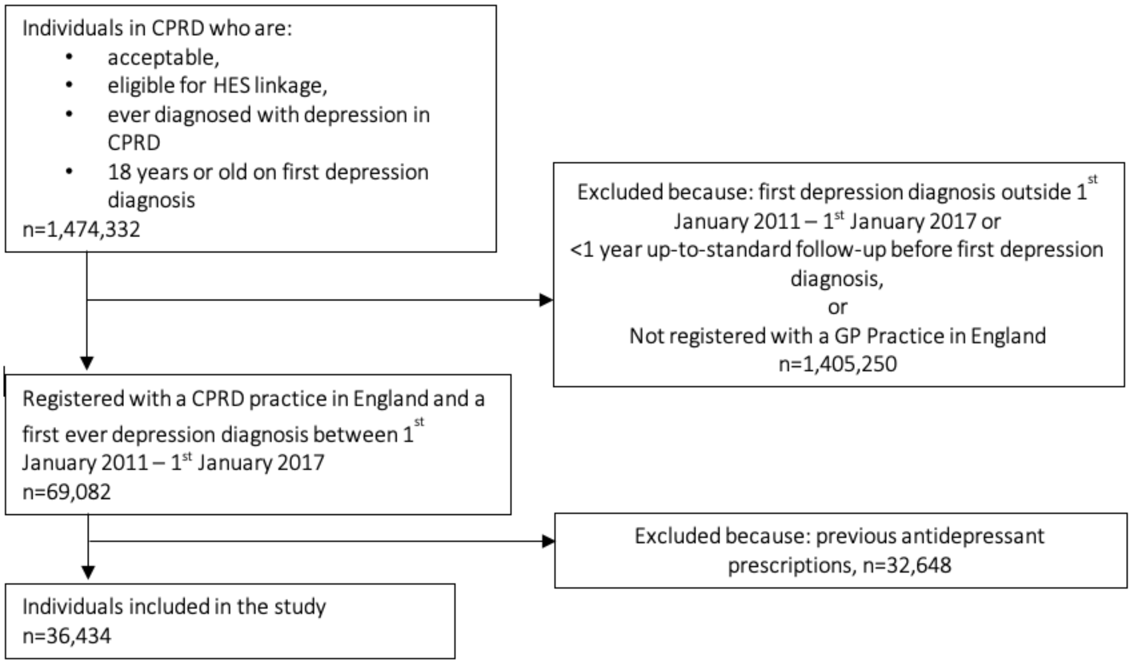

Abbreviations: CPRD – Clinical Practice Research Datalink; HES – Hospital Episode Statistics

Algorithms used to identify BMI and smoking status

Both smoking status and BMI were identified using an algorithm that utilised primary care records to identify the status recorded closest to the date of depression diagnosis. For BMI, Read codes for BMI category were not used because they are rarely recorded. Instead, BMI was calculated using height and weight measures recorded closest to the date of depression diagnosis. In the algorithm, records within -1 year to +1 month of the date of depression diagnosis were regarded as the best, +1 month to +1 year from the date of depression diagnosis as second best, the nearest prior to the year before the date of depression diagnosis as the third best, and within +1 year from the date of depression diagnosis as the worst. BMI was categorised as underweight (<18.5kg/m^2), normal (18.5 – <25.0 kg/m^2) or overweight (≥25.0 kg/m^2). Smoking status was classified as current smoker, ex-smoker, or non-smoker.

For peer review only - http://bmjopen.bmj.com/site/about/guidelines.xhtml

The RECORD statement – checklist of items, extended from the STROBE statement, that should be reported in observational studies using routinely collected health data.

	Item No.	STROBE items	Location in manuscript where items are reported	RECORD items	Location in manuscript where items are reported
**Title and abstract**					
	1	(a) Indicate the study's design with a commonly used term in the title or the abstract (b) Provide in the abstract an informative and balanced summary of what was done and what was found	1-22	RECORD 1.1: The type of data used should be specified in the title or abstract. When possible, the name of the databases used should be included.<br><br>RECORD 1.2: If applicable, the geographic region and timeframe within which the study took place should be reported in the title or abstract.<br><br>RECORD 1.3: If linkage between databases was conducted for the study, this should be clearly stated in the title or abstract.	1-2
**Introduction**					
Background rationale	2	Explain the scientific background and rationale for the investigation being reported	4		
Objectives	3	State specific objectives, including any prespecified hypotheses	4		
**Methods**					
Study Design	4	Present key elements of study design early in the paper	5		
Setting	5	Describe the setting, locations, and relevant dates, including periods of recruitment, exposure, follow-up, and data collection	5		

For peer review only

Participants	6	*(a) Cohort study* - Give the eligibility criteria, and the sources and methods of selection of participants. Describe methods of follow-up<br>*Case-control study* - Give the eligibility criteria, and the sources and methods of case ascertainment and control selection. Give the rationale for the choice of cases and controls<br>*Cross-sectional study* - Give the eligibility criteria, and the sources and methods of selection of participants<br><br>*(b) Cohort study* - For matched studies, give matching criteria and number of exposed and unexposed<br>*Case-control study* - For matched studies, give matching criteria and the number of controls per case	5	RECORD 6.1: The methods of study population selection (such as codes or algorithms used to identify subjects) should be listed in detail. If this is not possible, an explanation should be provided.<br><br>RECORD 6.2: Any validation studies of the codes or algorithms used to select the population should be referenced. If validation was conducted for this study and not published elsewhere, detailed methods and results should be provided.<br><br>RECORD 6.3: If the study involved linkage of databases, consider use of a flow diagram or other graphical display to demonstrate the data linkage process, including the number of individuals with linked data at each stage.	5-6 and supplementary material
Variables	7	Clearly define all outcomes, exposures, predictors, potential confounders, and effect modifiers. Give diagnostic criteria, if applicable.	5-6 and supplementary material	RECORD 7.1: A complete list of codes and algorithms used to classify exposures, outcomes, confounders, and effect modifiers should be provided. If these cannot be reported, an explanation should be provided.	5
Data sources/ measurement	8	For each variable of interest, give sources of data and details of methods of assessment (measurement).<br>Describe comparability of assessment methods if there is more than one group	5-6 and supplementary material		

For peer review only

Bias	9	Describe any efforts to address potential sources of bias	5-6		
Study size	10	Explain how the study size was arrived at	5-6		
Quantitative variables	11	Explain how quantitative variables were handled in the analyses. If applicable, describe which groupings were chosen, and why	5-6		
Statistical methods	12	(a) Describe all statistical methods, including those used to control for confounding<br>(b) Describe any methods used to examine subgroups and interactions<br>(c) Explain how missing data were addressed<br>(d) *Cohort study* - If applicable, explain how loss to follow-up was addressed<br>*Case-control study* - If applicable, explain how matching of cases and controls was addressed<br>*Cross-sectional study* - If applicable, describe analytical methods taking account of sampling strategy<br>(e) Describe any sensitivity analyses	6		
Data access and cleaning methods		..		RECORD 12.1: Authors should describe the extent to which the investigators had access to the database population used to create the study population.	5

For peer review only

				RECORD 12.2: Authors should provide information on the data cleaning methods used in the study.	
Linkage		..		RECORD 12.3: State whether the study included person-level, institutional-level, or other data linkage across two or more databases. The methods of linkage and methods of linkage quality evaluation should be provided.	5
**Results**					
Participants	13	(a) Report the numbers of individuals at each stage of the study (*e.g.*, numbers potentially eligible, examined for eligibility, confirmed eligible, included in the study, completing follow-up, and analysed)<br>(b) Give reasons for non-participation at each stage.<br>(c) Consider use of a flow diagram	Supplementary material	RECORD 13.1: Describe in detail the selection of the persons included in the study (*i.e.,* study population selection) including filtering based on data quality, data availability and linkage. The selection of included persons can be described in the text and/or by means of the study flow diagram.	Supplementary material
Descriptive data	14	(a) Give characteristics of study participants (*e.g.*, demographic, clinical, social) and information on exposures and potential confounders<br>(b) Indicate the number of participants with missing data for each variable of interest<br>(c) *Cohort study* - summarise follow-up time (*e.g.*, average and total amount)	7, 11-12		
Outcome data	15	*Cohort study* - Report numbers of outcome events or summary measures over time<br>*Case-control study* - Report numbers in each exposure	8-10		

For peer review only

		category, or summary measures of exposure<br>*Cross-sectional study* - Report numbers of outcome events or summary measures			
Main results	16	(a) Give unadjusted estimates and, if applicable, confounder-adjusted estimates and their precision (e.g., 95% confidence interval). Make clear which confounders were adjusted for and why they were included<br>(b) Report category boundaries when continuous variables were categorized<br>(c) If relevant, consider translating estimates of relative risk into absolute risk for a meaningful time period	11-12		
Other analyses	17	Report other analyses done—e.g., analyses of subgroups and interactions, and sensitivity analyses	N/A		
**Discussion**					
Key results	18	Summarise key results with reference to study objectives	13		
Limitations	19	Discuss limitations of the study, taking into account sources of potential bias or imprecision. Discuss both direction and magnitude of any potential bias	14	RECORD 19.1: Discuss the implications of using data that were not created or collected to answer the specific research question(s). Include discussion of misclassification bias, unmeasured confounding, missing data, and changing eligibility over time, as they pertain to the study being reported.	14-15
Interpretation	20	Give a cautious overall interpretation of results considering objectives,	15		

For peer review only

		limitations, multiplicity of analyses, results from similar studies, and other relevant evidence			
Generalisability	21	Discuss the generalisability (external validity) of the study results	15		
**Other Information**					
Funding	22	Give the source of funding and the role of the funders for the present study and, if applicable, for the original study on which the present article is based	17		
Accessibility of protocol, raw data, and programming code		..		RECORD 22.1: Authors should provide information on how to access any supplemental information such as the study protocol, raw data, or programming code.	17

*Reference: Benchimol EI, Smeeth L, Guttmann A, Harron K, Moher D, Petersen I, Sørensen HT, von Elm E, Langan SM, the RECORD Working Committee. The REporting of studies Conducted using Observational Routinely-collected health Data (RECORD) Statement. *PLoS Medicine* 2015; in press.

*Checklist is protected under Creative Commons Attribution (CC BY) license.

For peer review only

BMJ Open

Alcohol use recording in adults with depression in English primary care: a cross-sectional study

Journal:	*BMJ Open*
Manuscript ID	bmjopen-2021-055975.R1
Article Type:	Original research
Date Submitted by the Author:	26-Nov-2021
Complete List of Authors:	Adesanya, Elizabeth; LSHTM, Department of Non-Communicable Disease Epidemiology<br>Cook, Sarah; LSHTM, Department of Non-Communicable Disease Epidemiology<br>Crellin, Elizabeth; The Health Foundation<br>Langan, Sinead; London School of Hygiene & Tropical Medicine, Department of Non-Communicable Disease Epidemiology<br>Mansfield, Kathryn; LSHTM, Department of Non-Communicable Disease Epidemiology<br>Smeeth, Liam; LSHTM, Department of Non-Communicable Disease Epidemiology<br>Herrett, Emily; LSHTM, Department of Non-Communicable Disease Epidemiology
**Primary Subject Heading**:	Epidemiology
Secondary Subject Heading:	General practice / Family practice, Mental health, Epidemiology
Keywords:	EPIDEMIOLOGY, PRIMARY CARE, Depression & mood disorders < PSYCHIATRY, Substance misuse < PSYCHIATRY

SCHOLARONE™
Manuscripts

I, the Submitting Author has the right to grant and does grant on behalf of all authors of the Work (as defined in the below author licence), an exclusive licence and/or a non-exclusive licence for contributions from authors who are: i) UK Crown employees; ii) where BMJ has agreed a CC-BY licence shall apply, and/or iii) in accordance with the terms applicable for US Federal Government officers or employees acting as part of their official duties; on a worldwide, perpetual, irrevocable, royalty-free basis to BMJ Publishing Group Ltd ("BMJ") its licensees and where the relevant Journal is co-owned by BMJ to the co-owners of the Journal, to publish the Work in this journal and any other BMJ products and to exploit all rights, as set out in our licence.

The Submitting Author accepts and understands that any supply made under these terms is made by BMJ to the Submitting Author unless you are acting as an employee on behalf of your employer or a postgraduate student of an affiliated institution which is paying any applicable article publishing charge ("APC") for Open Access articles. Where the Submitting Author wishes to make the Work available on an Open Access basis (and intends to pay the relevant APC), the terms of reuse of such Open Access shall be governed by a Creative Commons licence – details of these licences and which Creative Commons licence will apply to this Work are set out in our licence referred to above.

Other than as permitted in any relevant BMJ Author's Self Archiving Policies, I confirm this Work has not been accepted for publication elsewhere, is not being considered for publication elsewhere and does not duplicate material already published. I confirm all authors consent to publication of this Work and authorise the granting of this licence.

For peer review only

Alcohol use recording in adults with depression in English primary care: a cross-sectional study

Authors: Elizabeth I. Adesanya,[1] Sarah Cook,[1,2] Elizabeth Crellin,[3] Sinéad M. Langan,[1] Kathryn E. Mansfield,[1] Liam Smeeth,[1] Emily Herrett[1]

Affiliations

[1] Department of Non-Communicable Disease Epidemiology, London School of Hygiene and Tropical Medicine, London, WC1E 7HT, United Kingdom

[2] National Heart and Lung Institute, Imperial College London, London, SW3 6LY, United Kingdom

[3] The Health Foundation, London, EC4Y 8AP, United Kingdom

Corresponding author

Elizabeth Adesanya, elizabeth.adesanya@lshtm.ac.uk

London School of Hygiene and Tropical Medicine, Keppel Street, London, WC1E 7HT, United Kingdom

Word count

Abstract: 269 words

Main text: 3714 words (excluding title page, abstract, tables, acknowledgements, contributions, and references)

Tables: 2

Figures: 2

Supplementary Tables/Figures: 2

For peer review only

ABSTRACT

Objectives

To investigate alcohol-use recording in people with newly diagnosed depression in English primary care, and individual characteristics associated with the recording of alcohol use.

Design

A population-based cross-sectional study.

Setting

Primary care data from English practices contributing to the UK Clinical Practice Research Datalink.

Participants

We included adults (18yrs+) diagnosed with depression between 1st January 2011 – 1st January 2017 without previous antidepressant use and at least one year of registration before diagnosis.

Primary and secondary outcome measures

We described the proportion of individuals with alcohol use and level of alcohol use recorded at four timepoints (the date of depression diagnosis, three months before or after depression diagnosis, 12 months before or after depression diagnosis, and any point pre- or post-depression diagnosis). We used logistic regression to investigate individual characteristics associated with alcohol-use recording in the three months before or after depression diagnosis.

Results

For peer review only - http://bmjopen.bmj.com/site/about/guidelines.xhtml

We identified 36,424 adults with depression. 538 (2%) had alcohol use recorded in the three months before or after depression diagnosis using formal validated methods such as the Alcohol Use Disorders Identification Test and its abbreviated versions. At each timepoint, most individuals with alcohol use recorded were low risk drinkers. Alcohol-use recording in the three months before or after depression diagnosis was associated with male sex (OR=1.38, 95% CI: 1.29,1.48) and several other individual-level factors.

Conclusions

Our study shows low levels of alcohol-use recording in the three months before or after depression diagnosis. Levels of alcohol-use recording varied depending on individual characteristics. Incentivised recording of alcohol use will increase completeness, which could improve clinical management and reduce missed opportunities for care in people with depression.

For peer review only

For peer review only - http://bmjopen.bmj.com/site/about/guidelines.xhtml

ARTICLE SUMMARY

Strengths and limitations of this study

- This study used CPRD data that is broadly representative of the UK population to provide generalisable, real world evidence of alcohol use recording in people with depression in English primary care.
- We used formal (i.e., the Alcohol Use Disorders Identification Test) and informal (i.e., units consumed per week) methods to identify and provide detailed descriptions of alcohol use in people with depression.
- Primary care records may not completely capture alcohol screening and alcohol usage, so our findings relating to recorded alcohol use may therefore underestimate both levels of alcohol screening and alcohol use among individuals with depression.
- Differences in coding practices between individual GPs and between different GP practices, and ambiguity in descriptions of alcohol use levels (e.g., whether they capture actual alcohol consumption in the previous week or typical weekly consumption) may have led to misclassification of alcohol use where it is recorded.

For peer review only

For peer review only - http://bmjopen.bmj.com/site/about/guidelines.xhtml

INTRODUCTION

Harmful alcohol use is a leading risk factor for premature death and disability.[1] In England, 22% of adults consume harmful levels of alcohol,[2] with alcohol-related harm costing the National Health Service (NHS) £3.5 billion annually.[3] Harmful alcohol use has been linked with the common mental health condition of depression.[4] In 2014, 20% of people aged 16 and over in the UK had symptoms of anxiety or depression.[5] The relationship between harmful alcohol use and depression is bidirectional – people with depression are more likely to drink harmfully, and, harmful levels of alcohol can act as a depressant, leading people who drink to excess experiencing symptoms of depression.[6,7] In people with depression, harmful alcohol consumption may exacerbate depression through several mechanisms: 1) alcohol can affect the adherence and effectiveness of depression treatment, worsening treatment outcomes;[8] 2) evidence suggests heavy alcohol use can directly lower serotonin levels (a hormone hypothesised to play a role in depression pathophysiology);[9,10] and 3) harmful alcohol use may worsen depressive symptoms by intensifying feelings of shame, guilt and low self-esteem, all of which are symptoms of depression.[9]

Screening for alcohol use allows early detection of harmful alcohol use and subsequent intervention before health issues become pronounced or irreversible.[11] UK National Institute for Health and Care Excellence (NICE) guidelines published in 2010 emphasise alcohol screening in primary care for subgroups at increased risk of alcohol abuse, including people with depression.[12] The Alcohol Use Disorders Identification Test (AUDIT) is a commonly used screening tool, and abbreviated versions have been developed for rapid screening in primary care and emergency departments (Fast Alcohol Screening Test [FAST], AUDIT for consumption [AUDIT C]).[13] Alcohol use is also recorded in primary care health records using other measures such as drinking status and level of alcohol consumption.[14,15]

For peer review only

Given the burden of harmful alcohol use and depression in the UK, and the damaging effects of harmful alcohol use in people with depression, it is crucial that we understand the extent to which alcohol use is recorded in people with depression. Previous work has examined alcohol-use recording generally in UK primary care,[14-16] but this is the first study to investigate alcohol-use recording specifically in people with newly diagnosed depression.

For peer review only

METHODS

Study design and setting

We identified adults (18+years) with depression using prospectively collected primary care electronic health record data from the UK Clinical Practice Research Datalink (CPRD) GOLD database,[17] and linked Index of Multiple Deprivation (IMD) data.[18] The CPRD is an ongoing, nationwide primary care database of routinely collected and anonymised medical records.[17] We included all adults (≥18 years) in the CPRD GOLD database registered with a CPRD practice in England with a first depression diagnosis recorded between 1st January 2011 and 1st January 2017. We excluded individuals if they had less than one year of research standard registration prior to their depression diagnosis, or they had previously been prescribed antidepressants (to better capture newly diagnosed depression).

Factors associated with recording of alcohol use

We investigated a range of potential individual-level factors that could be associated with alcohol-use recording in primary care for people with depression: age at depression diagnosis, sex, ethnicity, deprivation, geographical region, smoking status, body mass index (BMI), the number of years after GP registration that depression was diagnosed (1-2 years, 2-3 years, >3 years), depression management with medication (antidepressant classes: tricyclic antidepressants [TCAs], selective serotonin uptake inhibitors [SSRIs], monoamine oxidase inhibitors [MAOIs], and other antidepressants) or without medication (talking therapies, e.g., cognitive behavioural therapy), and selected comorbidities related to alcohol use (liver disease, hypertension, diabetes mellitus, anxiety and substance abuse).

We defined deprivation using quintiles of the Index of Multiple Deprivation (IMD). We identified liver disease, hypertension and diabetes mellitus based on the presence of a

For peer review only - http://bmjopen.bmj.com/site/about/guidelines.xhtml

recorded (morbidity-coded) diagnosis in primary care prior to the date of the first depression diagnosis. We identified anxiety and substance abuse based on the presence of a (morbidity-coded) diagnosis up to five years prior to the date of the first depression diagnosis. We used existing code lists and algorithms to define BMI, ethnicity, and smoking status.[19,20] For BMI and smoking status, we used the status recorded closest to the date of the first depression diagnosis. We identified depression management with medication using primary care prescription data recorded after the first ever depression diagnosis. We identified talking therapies based on the presence of morbidity codes for psychological therapies (e.g., cognitive behavioural therapy, counselling). We have made all code lists used in this study available to download from online repositories.[21,22] Algorithms used to identify BMI and smoking status are described in further detail in the supplementary material.

Alcohol use recording

We identified recorded alcohol use using primary care morbidity coding (Read codes) and data captured in structured data-entry fields. Records of alcohol use included:

1. Codes indicating the use of AUDIT, AUDIT-C or FAST screening and their associated scores.
2. Codes indicating the diagnosis of alcohol use disorders (AUDs).
3. Codes quantifying alcohol use:
    a. Drinking status (current, non, ex).
    b. Drinking level (Read coded as light, moderate, heavy, or non).
    c. Units consumed per week (none, 1-14, 15-42, 43+).

For peer review only - http://bmjopen.bmj.com/site/about/guidelines.xhtml

We categorised alcohol use recording in terms of: (1) Type: whether it was formal (recorded using formal, validated methods, i.e., AUDIT, AUDIT-C or FAST), or informal (i.e., morbidity codes suggesting individuals were asked about drinking status or alcohol intake, or had an AUD diagnosis); and (2) Timing: of recorded alcohol use in relation to depression diagnosis at four time points (date of depression diagnosis; in the three months before or after depression diagnosis; in the 12 months before or after depression diagnosis; or at any time pre-or post-depression diagnosis).

Statistical analysis

We described the prevalence and level of recorded alcohol use in individuals diagnosed with depression according to whether it was: (1) formal or informal; and (2) timing in relation to date of depression diagnosis. Individuals whose alcohol use was recorded using formal, validated screening tools (e.g., AUDIT) had their level of alcohol use described according to the recorded screening tool scores. Individuals whose alcohol use was recorded using informal methods (e.g., drinking status or alcohol consumption) had their level of alcohol use based on the recorded information (e.g., units per week). Individuals in the study may have been recorded more than once at each timepoint and using more than one method of alcohol use recording. We used logistic regression to estimate odds ratios (adjusted for age, sex, ethnicity, and deprivation) for the association between the factors potentially associated with alcohol-use recording in the three months before or after depression diagnosis. Individuals with missing data on any of the variables considered were excluded from regression analyses. Data were analysed using Stata V.15 (StataCorp, Texas, USA).

Patient and public involvement

Patients or the public were not involved in the design or analysis of this study.

For peer review only - http://bmjopen.bmj.com/site/about/guidelines.xhtml

For peer review only

For peer review only

For peer review only - http://bmjopen.bmj.com/site/about/guidelines.xhtml

RESULTS

Study population

We identified 36,434 adults with a depression diagnosis recorded between 1st January 2011 and 1st January 2017 who were eligible for study inclusion **(Supplementary Figure 1)**. Fifty-four percent (n=19,604) of the study population were female, and the median age was 38 (IQR=27-51). There was an approximately even distribution of the study population across the deprivation quintiles.

Prevalence of alcohol use recording and level of alcohol use among those recorded

31,007 (85%) individuals had their alcohol use recorded at any point pre-or post-depression diagnosis. However, only 6,012 (17%) had their alcohol use recorded in the three months before or after their depression diagnosis **(Figure 1)**.

Formal alcohol use recording

Alcohol use recording using formal validated methods (AUDIT, AUDIT-C or FAST) was low; 23% (n=8,284) of the study population had their alcohol use recorded at any point pre- or post-depression diagnosis, and only 2% (n=538) had formal alcohol screening recorded in the three months before or after depression diagnosis **(Table 1)**. Most individuals who had their alcohol use recorded at each of the timepoints using AUDIT or FAST were identified as low risk drinkers **(Table 1)**. In the three months before or after depression diagnosis, 17% (63/370) of people who had their alcohol use recorded using AUDIT, and 17% (20/116) of those recorded using FAST, were identified as harmful drinkers (i.e., score of ≥8 on AUDIT tool, and ≥3 on FAST tool). In the three months before or after depression diagnosis, 18% (67/370) of those with their alcohol use recorded using AUDIT, and 19% recorded using FAST (22/116), had invalid scores recorded (i.e., score of >40 on AUDIT tool, and >16 on

For peer review only - http://bmjopen.bmj.com/site/about/guidelines.xhtml

FAST tool). Over 90% of individuals at each of the four timepoints who were screened with AUDIT-C did not have a score recorded by their GP.

Informal alcohol use recording

Sixteen percent (n=5,724) of the study population had their alcohol use recorded using informal methods (i.e., codes suggesting that individuals were asked about drinking status or alcohol intake, or had an AUD diagnosed) in the three months before or after depression diagnosis, and 83% (n=30,175) of individuals were recorded at any point pre- or post-depression diagnosis **(Table 2)**. Of the 51% (n=2,915) of individuals who had their weekly alcohol consumption recorded (a method of informal alcohol use recording) in the three months before or after depression diagnosis, 56% (n=1,621) were low risk drinkers, while 37% (n=1,087) were harmful drinkers (defined as consuming more than the recommended 14 units a week for men and women). Twenty-five percent (n=6,818) of individuals who had their weekly alcohol consumption recorded (in units per week) at any point pre-or post-depression diagnosis were harmful drinkers.

Factors associated with alcohol use recording

We saw evidence of increased odds of alcohol use recording using both formal validated methods (AUDIT, AUDIT-C and FAST) and informal methods (drinking level, alcohol consumption, drinking status) in the three months before or after depression diagnosis after adjusting for age, sex, ethnicity, and deprivation in: men, people living in the North of England compared to those living in the South, current or ex-smokers, individuals with their depression managed through talking therapies compared to those that go without, and people with hypertension or diabetes **(Figure 2, Supplementary Table 1)**. There was also evidence of linear associations between deprivation, time since GP registration and alcohol

For peer review only - http://bmjopen.bmj.com/site/about/guidelines.xhtml

use recording. The least deprived individuals were more likely to have their alcohol use recorded than the most deprived individuals. Individuals registered at their GP practice for more than three years had higher odds of alcohol use recording compared to those registered between 1-2 years. There was strong evidence of a non-linear association ($p<0.001$) between alcohol use recording and age at depression diagnosis. Individuals aged between 60-69 had more than twice the odds of alcohol use recording compared to those aged 18-29.

For peer review only

Table 1: Number of individuals with alcohol use recorded using formal validated methods (AUDIT, AUDIT-C or FAST) at pre-specified timepoints in relation to depression diagnosis (data are N(%) unless otherwise specified)

	Timing of alcohol-use recording in relation to depression diagnosis N (%)			
	**On date of depression diagnosis**	**+/- 3 months**	**+/- 12 months**	**Any point**
**Formal alcohol use recording (any) [a]**	**93 (0.3)**	**538 (1.5)**	**1,515 (4.2)**	**8,284 (22.7)**
**AUDIT [b]**	**68 (73.1)**	**370 (68.8)**	**1,029 (67.9)**	**4,615 (55.7)**
Low risk (AUDIT score 0-7)	34 (50.0)	240 (64.9)	739 (71.8)	3,193 (69.2)
Hazardous (AUDIT score 8-15)	11 (16.2)	43 (11.6)	95 (9.2)	318 (6.9)
High risk (AUDIT score 16-19)	*	7 (1.9)	10 (1.0)	27 (0.6)
Possible dependence (AUDIT score 20-40)	*	13 (3.5)	18 (1.8)	37 (0.8)
Invalid score value recorded (AUDIT score >40)	17 (25.0)	67 (18.1)	167 (16.2)	1,040 (22.5)
**AUDIT-C [b]**	**22 (23.7)**	**139 (25.8)**	**395 (26.1)**	**2,886 (34.8)**
Low risk (AUDIT-C score 0-4)	0 (0.0)	10 (7.2)	22 (5.6)	79 (2.7)
Hazardous (AUDIT-C score 5-7)	0 (0.0)	*	*	19 (0.7)
High risk (AUDIT-C score 8-10)	0 (0.0)	0 (0.0)	*	6 (0.2)
Possible dependence (AUDIT-C score 11-12)	0 (0.0)	0 (0.0)	0 (0.0)	0 (0.0)
AUDIT-C score not recorded	22 (100.00)	128 (92.1)	366 (92.7)	2,782 (96.4)
**FAST [b]**	**16 (17.2)**	**116 (21.6)**	**368 (24.3)**	**2,849 (34.4)**
Low risk (FAST score 0-2)	11 (68.8)	74 (63.8)	242 (65.8)	1,633 (57.3)
Hazardous (FAST score 3-16)	*	20 (17.2)	48 (13.0)	336 (11.8)
Invalid score value recorded (FAST score >16)	*	22 (19.0)	78 (21.2)	880 (30.9)

For peer review only - http://bmjopen.bmj.com/site/about/guidelines.xhtml

Abbreviations: +/- 3 months – alcohol use recorded in the 3 months before or after depression diagnosis; +/- 12 months – alcohol use recorded in the year before or after depression diagnosis; any point – alcohol use recorded at any point pre- or post-depression diagnosis

[a] Percentage calculated out of the total number of individuals in the cohort (36,434)

[b] Percentage calculated out of the total number of individuals with alcohol use recorded using formal validated methods – individuals may have more than one type of formal recording, so numbers add to >100%; risk scores represent the proportion at risk identified using that particular test

[*] Cell counts less than 5 suppressed to preserve individuals' confidentiality.

For peer review only

Table 2: Number of individuals with alcohol use recorded using informal methods (i.e., alcohol status, level of alcohol use) at pre-specified timepoints in relation to depression diagnosis (data are N(%) unless otherwise specified)

	Timing of alcohol-use recording in relation to depression diagnosis N (%)			
	Date of depression diagnosis	+/- 3 months	+/- 12 months	Any point
Informal alcohol use recording (any) [a]	2,039 (5.6)	5,724 (15.7)	11,361 (31.2)	30,175 (82.8)
**Recording of current alcohol status [b]**	**901 (44.2)**	**3,025 (52.9)**	**6,643 (58.5)**	**24,195 (80.2)**
Non	313 (34.7)	947 (31.3)	2,081 (31.3)	8,145 (33.7)
Current	496 (55.1)	1,777 (58.7)	3,907 (58.8)	14,198 (58.7)
Ex	92 (10.2)	301 (10.0)	655 (9.9)	1,852 (7.7)
**Recording of codes indicating level of alcohol use [b]**	**855 (41.9)**	**2,893 (50.5)**	**6,406 (56.4)**	**24,377 (80.8)**
None	405 (47.4)	1,239 (42.8)	2,675 (41.8)	9,370 (38.4)
Light	304 (35.6)	1,260 (43.6)	2,956 (46.1)	11,383 (46.7)
Moderate	36 (4.2)	123 (4.3)	282 (4.4)	2,106 (8.6)
Heavy	110 (12.9)	271 (9.4)	493 (7.7)	1,518 (6.2)
**Recording of units of alcohol consumed per week [b]**	**1,090 (53.5)**	**2,915 (50.9)**	**6,390 (56.2)**	**27,377 (90.7)**
None	78 (7.2)	207 (7.1)	474 (7.4)	3,744 (13.7)
1-14	499 (45.8)	1,621 (55.6)	3,865 (60.5)	16,815 (61.4)
15-42	322 (29.5)	745 (25.6)	1,498 (23.4)	5,477 (20.0)
43+	191 (17.5)	342 (11.7)	553 (8.7)	1,341 (4.9)

For peer review only - http://bmjopen.bmj.com/site/about/guidelines.xhtml

**Recording of codes relating to alcohol use disorders [b]**	**39 (1.9)**	**117 (2.0)**	**213 (1.9)**	**584 (1.9)**

Abbreviations: +/- 3 months – alcohol use recorded in the 3 months before or after depression diagnosis; +/- 12 months – alcohol use recorded in the year before or after depression diagnosis; any point – alcohol use recorded at any point pre- or post-depression diagnosis

[a] Percentage calculated out of the total number of individuals in the cohort (36,434)

[b] Percentage calculated out of the total number of individuals with alcohol use recorded using informal methods – individuals may have more than one type of informal recording, so numbers add to >100%; percentages are out of the number of people with alcohol use recording using that specific method

For peer review only

DISCUSSION

Summary

We found that levels of alcohol-use recording in people with depression were high: 85% of individuals had their alcohol use recorded using either formal or informal methods at any point pre-or post-depression diagnosis. However, only 17% of individuals had their alcohol use recorded in the three months before or after depression diagnosis, with only 2% of these individuals recorded using formal validated tools (AUDIT, AUDIT-C or FAST). Just over one-third (37%) of individuals who had their weekly alcohol consumption recorded in the three months before or after depression diagnosis were harmful drinkers (defined as consuming more than the recommended 14 units a week for men and women), whereas 25% of individuals who had their weekly alcohol consumption recorded at any point pre- or post-depression diagnosis were harmful drinkers.

After adjusting for age, sex, ethnicity, and deprivation, we found that several individual-level characteristics (including living in the least deprived areas, current or ex-smokers, and comorbid hypertension or diabetes) were associated with alcohol-use recording in the three months before or after depression diagnosis.

Comparison with existing literature

Compared to a 2019 study, which showed that 52% of people in UK primary care had a record of alcohol use,[14] our study suggests that alcohol use recording in people with depression was higher (85%). Studies using primary care data in the UK and US have also provided evidence that individuals with depression or other psychiatric conditions are more likely to have their alcohol use recorded or screened for an alcohol use disorder compared to the general population.[14,23]

For peer review only - http://bmjopen.bmj.com/site/about/guidelines.xhtml

According to data from the 2018 Health Survey for England (HSE), 22% of all adults in England consumed more than the recommended 14 units a week.[2] This is comparable to the figure (25%) in the present study among those with alcohol consumption recorded at any time. However, the proportion of harmful drinkers identified by the HSE is lower than the proportion of individuals with depression in this study who had their alcohol consumption recorded in the three months before or after depression diagnosis and were found to be harmful drinkers (37%).

Our findings regarding are comparable to studies in Sweden where the proportion of individuals with depression that consumed harmful amounts of alcohol ranged from 21-23% (compared to 25% in our study).[24,25] However, our findings regarding harmful weekly alcohol consumption are dissimilar to studies conducted in: 1) Singapore, where 19% of people with depression were harmful drinkers;[26] and 2) South Korea, where 51% of people with depression were harmful drinkers.[27] GP recording practice, as well as cultural and governmental variations, are possible explanations of this difference in results. Lower levels of harmful drinking in Singapore may be explained by strict alcohol laws; alcohol cannot be sold or consumed in public from 10:30pm to 7:00am.[26] Similarly, the prominent drinking culture in South Korea may contribute to the increased prevalence.[28]

Our study found that people with depression who were men, ex/current smokers, aged 60-69, diagnosed with diabetes or hypertension, from areas with the lowest level of deprivation, or who lived in the North of England were more likely to have their alcohol use recorded compared to other people with depression. Other UK primary care studies of alcohol-use recording have also demonstrated increased rates of alcohol-use recording in people with diabetes and hypertension, and those aged 60 to 69 years.[14-16] There is also evidence to suggest that some of the factors we identified as being linked to alcohol-use recording are

For peer review only - http://bmjopen.bmj.com/site/about/guidelines.xhtml
For peer review only

linked to harmful alcohol use. For example, smokers and ex-smokers have been shown to be more likely to consume harmful amounts of alcohol when compared to non-smokers.[29] Data from the 2018 HSE also showed that men were 15% more likely to drink harmfully compared to women, and the highest proportion of heavier drinkers was found in the North of England.[2] This evidence from HSE taken alongside our results may suggest that GPs are more likely to record alcohol use in those perceived to be at higher risk even amongst those with depression.

However, some characteristics that have been identified as being associated with increased levels of alcohol-use recording in our study contrast with finding from other UK studies. Other studies have shown that women are more likely to have their alcohol use recorded compared to men,[15] and those in the most deprived regions are more likely to be have recorded alcohol use than those in the least deprived areas.[14,15] However, the increased odds of alcohol use recording in the least deprived areas seen in this study may be explained by GP practices in more deprived areas possibly having fewer resources to deliver timely and formal alcohol-use recording.

Strengths and limitations

The CPRD GOLD dataset is broadly representative of the UK population with regards to age and sex,[15] and consequently our results can be broadly generalised to the rest of the UK population. Our study was able to use complete data on key variables such as age, sex, deprivation and comorbid conditions. Importantly this study captured levels of alcohol-use recording in a real life setting in UK primary care, and therefore highlights potential missed opportunities for care in people with depression.

For peer review only - http://bmjopen.bmj.com/site/about/guidelines.xhtml
For peer review only

An important limitation of this study is that individuals without alcohol use recorded may have discussed alcohol with their GP, but this was not documented, leading to underestimation of alcohol-use screening based on recording as a proxy marker. Lack of documentation may be more likely where low levels of drinking were reported. While we cannot directly extrapolate our findings to discussion of alcohol use within GP consultations, our findings related to recording are important given documentation of alcohol use is crucial for informing future management and monitoring change in drinking.

Similarly, although our analysis found an association between various factors and alcohol-use recording, GPs may record alcohol use in individuals who they believe are at higher risk of harmful alcohol use for reasons that are not in their coded medical record. GPs may also base their decisions to ask about, and record, alcohol use on the previous alcohol level recorded. For instance, people who had previously had high levels of alcohol use recorded may be more likely to have their alcohol use recorded when they are diagnosed with depression. Equally the decision to record alcohol use if discussed in the consultation may also be related to the level of alcohol use (GPs may be more likely to document harmful drinking) which could influence the estimates of harmful drinking among those with depression found here.

Another limitation is the potential imprecision of alcohol consumption data recorded in primary care. A number of factors related to recording mean that our estimates may be an unreliable estimate of true alcohol intake. Firstly, we noted that some people with alcohol use recorded using formal methods either did not have a score for the test recorded (particularly frequent in those with alcohol use recorded using AUDIT-C), or the recorded score was outside the total possible value for the test. It is unclear how such missing data would affect our findings. Secondly, it is unclear whether individuals with recorded alcohol

For peer review only - http://bmjopen.bmj.com/site/about/guidelines.xhtml

use were asked about recent consumption (how much they actually consumed in the past week), or typical consumption (how much they typically consume in a week). The potential ambiguity in the consumption data implies differences in coding practices between individual GPs and between different GP practices. It also suggests that consumption records need to be used cautiously for research as they may not necessarily be capturing the same thing for each individual. And finally, individuals may be less truthful about their alcohol consumption if asked face-to-face when compared to a self-completed questionnaire (potentially underestimating harmful-drinking levels).

Data on variables such as substance abuse and depression management without medication are likely to be poorly captured within the CPRD. Restricting entry into the study to individuals without previous antidepressant prescriptions allowed us to more cleanly capture people with newly diagnosed depression, but it also reduced sensitivity and we may have missed individuals that had depression who had been prescribed antidepressants before depression diagnosis was coded.

Implication for research and/or practice

Our findings have important implications for alcohol use recording among people with depression in English primary care. Our results indicate that GPs are not recording alcohol use in people with depression as effectively as they could be (in terms of capturing all individuals or using the most appropriate tools). A lack of recording does not mean that appropriate alcohol-use screening is not happening in practice. However, the low levels of alcohol-use screening recording we saw may suggest that NICE guidelines[12] (recommending alcohol-use screening in people with depression) have not been closely followed in UK primary care, suggesting that alcohol use may not have been discussed and

For peer review only - http://bmjopen.bmj.com/site/about/guidelines.xhtml
For peer review only

addressed by the GP in a timely manner. Not screening for alcohol-use represents a potential missed opportunity for care in people with depression that may lead to poorer health outcomes and may highlight an area for improvement within primary care.

While it is encouraging that some alcohol use recording is taking place, it is imperative that all individuals with depression or those at high risk of alcohol misuse have their alcohol use routinely screened in primary care using quick validated tools such as the AUDIT-C or FAST. While these questionnaires are built-in to most GP clinical systems in the UK, more effective and efficient alcohol-use screening might be possible through self-administered questionnaires (simply administered in GP waiting rooms or using online tools). Routine screening leads to early detection and the opportunity for GPs to provide brief alcohol interventions that have been proven to be effective at reducing alcohol consumption among people who are drinking at harmful levels.[30]

Evidence shows that financial incentives can increase primary care alcohol use recording.[30] The UK government recently introduced an, 'Alcohol related risk reduction scheme' requiring GPs to identify newly registered individuals aged 16 or over who drink harmful levels of alcohol, however there is no emphasis on depression nor funding attached.[31] There is also currently no financial incentive for recording alcohol use in people with depression through the Quality and Outcomes Framework (QOF),[32] even though in 2019, a NICE suggested a QOF indicator to record the percentage of individuals diagnosed with depression in the last year who had been screened for hazardous drinking in the three months before or after their depression diagnosis.[33] Additionally, the small financial reward offered to practices in England for screening newly registered adults for harmful alcohol use as part of Clinical Directed Enhanced Services (DES) ceased in 2015.[34,35]

For peer review only - http://bmjopen.bmj.com/site/about/guidelines.xhtml

The incentivisation of alcohol use recording may help to increase numbers of people recorded, which is crucial for future patient management and will improve holistic care for individuals with depression. Adding alcohol-use recording as a QOF indicator or rewarding services for recording of alcohol use is likely to improve clinical practice and patient outcomes.

For peer review only

For peer review only - http://bmjopen.bmj.com/site/about/guidelines.xhtml

DECLARATIONS AND ACKNOWLEDGEMENTS

Contributions

EH, SC and KEM designed the study. EC and EH extracted the data. EIA performed the analysis and wrote the first draft of the manuscript. All authors (EIA, SC, EC, SML, KEM, LS and EH) reviewed and approved the final manuscript. Dr Alasdair Henderson contributed to the development of Figure 2 in the final manuscript.

Acknowledgements

This study is based in part on data from the Clinical Practice Research Datalink obtained under licence from the UK Medicines and Healthcare products Regulatory Agency. The data is provided by patients and collected by the NHS as part of their care and support. The interpretation and conclusions contained in this study are those of the authors alone. The study was approved by the CPRD's Independent Scientific Advisory Committee (Protocol number: 19_077).

Funding

EH is funded by a post-doctoral fellowship from the NIHR (Reference: PDF-2016-09-029). SML is funded by a Wellcome Trust Senior Clinical Fellowship in Science (Reference: 205039/Z/16/Z). For the purpose of Open Access, the author has applied a CC BY public copyright licence to any Author Accepted Manuscript (AAM) version arising from this submission.

Ethical approval

The study was approved by the London School of Hygiene and Tropical Medicine Research Ethics Committee and by the Clinical Practice Research Datalink Independent Scientific Advisory Committee (ISAC Protocol Number: 19_077).

For peer review only

Competing interests

None declared

Data statement

Data were obtained from a third party and are not publicly available.

For peer review only

REFERENCES

1. World Health Organisation (WHO). *Alcohol*. Available from: https://www.who.int/health-topics/alcohol#tab=overview [Accessed 29th January 2020].

2. NHS Digital. *Health Survey for England (HSE) 2018*. Available from: https://digital.nhs.uk/data-and-information/publications/statistical/health-survey-for-england/2018 [Accessed 20th November 2020].

3. Institute of Alcohol Studies (IAS). *Estimates of the cost of alcohol*. Available from: http://www.ias.org.uk/Alcohol-knowledge-centre/Economic-impacts/Factsheets/Estimates-of-the-cost-of-alcohol.aspx [Accessed 20th November 2020].

4. Public Health England (PHE). *Health Matters: Harmful Drinking and Alcohol Dependence*. Available from: https://www.gov.uk/government/publications/health-matters-harmful-drinking-and-alcohol-dependence/health-matters-harmful-drinking-and-alcoholdependence#the-scale-of-the-problem [Accessed 29th January 2020].

5. Mental Health Foundation. *Fundamental Facts about Mental Health 2016*. Available from: https://www.mentalhealth.org.uk/file/2518/download?token=RU6lzcSK [Accessed 29th January 2020].

6. Boden JM, Fergusson DM. Alcohol and depression. *Addiction*. 2011;106(5):906-14. doi: 10.1111/j.1360-0443.2010.03351.x.

7. Ramsey SE, Engler PA, Stein MD. Alcohol Use Among Depressed Patients: The Need for Assessment and Intervention. *Prof Psychol Res Pr*. 2005;36(2):203-207. doi: 10.1037/0735-7028.36.2.203.

8. Hjorthøj C, Østergaard ML, Benros ME, et al. Association between alcohol and substance use disorders and all-cause and cause-specific mortality in schizophrenia, bipolar disorder, and unipolar depression: a nationwide, prospective, register-based study. *Lancet Psychiatry*. 2015;2(9):801–8. doi: 10.1016/S2215-0366(15)00207-2.

9. Ramsey SE, Engler PA, Stein MD. Alcohol Use Among Depressed Patients: The Need for Assessment and Intervention. *Prof Psychol Res Pr*. 2005;36(2):203-207. doi: 10.1037/0735-7028.36.2.203.

10. Cowen PJ, Browning M. What has serotonin to do with depression? *World Psychiatry*. 2015;14(2):158-60. doi: 10.1002/wps.20229.

For peer review only - http://bmjopen.bmj.com/site/about/guidelines.xhtml

11. World Health Organisation (WHO). *Screening and brief intervention for alcohol problems in primary health care*. Available from: https://www.who.int/substance_abuse/activities/sbi/en/ [Accessed 29th January 2020].

12. National Institute for Health and Care Excellence (NICE). *Alcohol-use disorders: prevention.* Available from: https://www.nice.org.uk/guidance/ph24/resources/alcoholuse-disorders-prevention-pdf-1996237007557 [Accessed 29th January 2020].

13. Reinert DF, Allen JP. The Alcohol Use Disorders Identification Test: An Update of Research Findings. *Alcohol Clin Exp Res*. 2007;31(2):185–99. doi: 10.1111/j.1530-0277.2006.00295.x.

14. Mansfield K, Crellin E, Denholm R, et al. Completeness and validity of alcohol recording in general practice within the UK: a cross-sectional study. *BMJ Open*. 2019;26;9(11):e031537. doi: 10.1136/bmjopen-2019-031537.

15. Khadjesari Z, Marston L, Petersen I, et al. Alcohol consumption screening of newly-registered patients in primary care: a cross-sectional analysis. *Br J Gen Pract.* 2013;63(615):706–12. doi: 10.3399/bjgp13X673720

16. Cheeta S, Drummond C, Oyefeso A, et al. Low identification of alcohol use disorders in general practice in England. *Addiction*. 2008;103(5):766-73. doi: 10.1111/j.1360-0443.2008.02198.x.

17. Herrett E, Gallagher AM, Bhaskaran K, et al. Data Resource Profile: Clinical Practice Research Datalink (CPRD). *Int J Epidemiol*. 2015;44(3):827–36. doi: 10.1093/ije/dyv098.

18. Department for Communities and Local Government. *The English Index of Multiple Deprivation (IMD) 2015 – Guidance*. Available from: https://assets.publishing.service.gov.uk/government/uploads/system/uploads/attachment_data/file/464430/English_Index_of_Multiple_Deprivation_2015_-_Guidance.pdf [Accessed 29th January 2020].

19. Mathur R, Bhaskaran K, Chaturvedi N, et al. Completeness and usability of ethnicity data in UK-based primary care and hospital databases. *J Public Health (Oxf)*. 2014;36(4):684-692. doi: 10.1093/pubmed/fdt116.

20. Bhaskaran K, Forbes HJ, Douglas I, et al. Representativeness and optimal use of body mass index (BMI) in the UK Clinical Practice Research Datalink (CPRD). *BMJ Open*. 2013;3: e003389. doi: 10.1136/bmjopen-2013-003389.

For peer review only - http://bmjopen.bmj.com/site/about/guidelines.xhtml

21. Adesanya E, Cook S, Mansfield KE, et al. Code lists for "Alcohol use recording in people with depression in English primary care". LSHTM Data Compass. London, United Kingdom: London School of Hygiene & Tropical Medicine; . Available from: https://doi.org/10.17037/DATA.00002183.

22. Herrett E, Cook S, Mansfield KE, et al. Code lists for "Completeness and validity of alcohol recording in general practice within the UK: a cross-sectional study". LSHTM Data Compass. London, United Kingdom: London School of Hygiene & Tropical Medicine; . Available from: https://doi.org/10.17037/data.00001071.

23. Edlund MJ, Unützer J, Wells KB. Clinician screening and treatment of alcohol, drug, and mental problems in primary care: results from healthcare for communities. *Med Care*. 2004;42(12):1158-66. doi: 10.1097/00005650-200412000-00002.

24. Eberhard S, Nordström G, Höglund P, Ojehagen A. Secondary prevention of hazardous alcohol consumption in psychiatric out-patients: a randomised controlled study. *Soc Psychiatry Psychiatr Epidemiol*. 2009;44(12):1013-21. doi: 10.1007/s00127-009-0023-7.

25. Åhlin J, Hallgren M, Öjehagen A, Källmén H, Forsell Y. Adults with mild to moderate depression exhibit more alcohol related problems compared to the general adult population: a cross sectional study. *BMC Public Health*. 2015;15(542). https://doi.org/10.1186/s12889-015-1837-8

26. Subramaniam M, Mahesh MV, Peh CX, et al. Hazardous alcohol use among patients with schizophrenia and depression. *Alcohol*. 2017;65:63-69. doi: 10.1016/j.alcohol.2017.07.008.

27. Park SC, Lee SK, Oh HS, et al. Hazardous drinking-related characteristics of depressive disorders in Korea: The CRESCEND study. *J Korean Med Sci*. 2014;30(1):74-81. doi: 10.3346/jkms.2015.30.1.74.

28. Ryu SY, Crespi CM, Maxwell AE. Drinking patterns among Korean adults: Results of the 2009 Korean community health survey. *J Prev Med Public Health*. 2013;46(4):183-191. doi: 10.3961/jpmph.2013.46.4.183.

29. Chiolero A, Wietlisbach V, Ruffieux C, et al. Clustering of risk behaviours with cigarette consumption: A population-based study. *Prev Med*. 2006;42(5):348-353. doi: https://doi.org/10.1016/j.ypmed.2006.01.011.

For peer review only

30. Hamilton FL, Laverty AA, Gluvajic D, et al. Effect of financial incentives on delivery of alcohol screening and brief intervention (ASBI) in primary care: longitudinal study. *J Public Health (Oxf)*. 2014;36(3):450-9. doi: 10.1093/pubmed/fdt121.

31. NHS Digital. *Alcohol related risk reduction scheme*. Available from: https://digital.nhs.uk/services/general-practice-gp-collections/service-information/alcohol-related-risk-reduction-scheme-2017-18#top [Accessed 29th January 2020].

32. NHS Employers. *Quality and Outcomes Framework: Guidance for GMS contract 2019/20*. Available from: https://www.england.nhs.uk/wp-content/uploads/2019/05/gms-contract-qof-guidance-april-2019.pdf [Accessed 29th January 2020].

33. National Institute for Health and Care Excellence (NICE). *Quality and Outcomes Framework indicator*. Available from: https://www.nice.org.uk/standards-and-indicators/qofindicators/the-percentage-of-patients-with-a-new-diagnosis-of-depression-or-anxiety-in-the-preceding-12-months-who-have-been-screened-for-unsafe-drinking-using-the-fast-or-audit-c-tool-in-the-3-months-before-or-after-their-diagnosis-being-recorded [Accessed 29th January 2020].

34. NHS Employers. *Clinical directed enhanced services (DES) guidance for GMS contract 2008/09*. Available from: https://www.lmc.org.uk/visageimages/guidance/2008/clinicaldesguidance.pdf [Accessed 29th January 2020].

35. Croner-i. *Enhanced Services: In-depth*. Available from: https://app.croneri.co.uk/topics/enhanced-services/indepth [Accessed 29th January 2020]

For peer review only

For peer review only - http://bmjopen.bmj.com/site/about/guidelines.xhtml

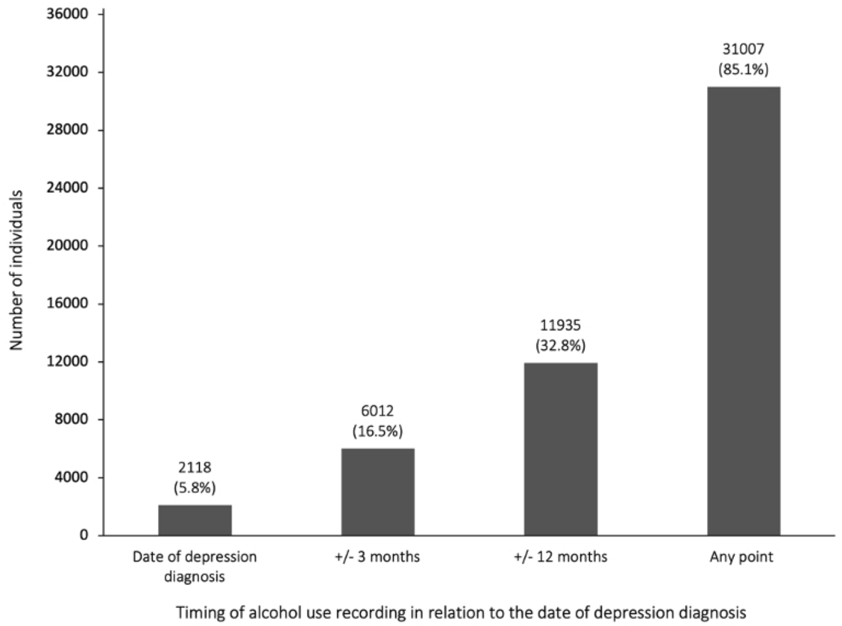

Abbreviations: +/- 3 months – alcohol use recorded in the three months before or after depression diagnosis; +/- 12 months – alcohol use recorded in the year before or after depression diagnosis; any point – alcohol use recorded at any point pre- or post-depression diagnosis

Prevalence of alcohol use recording at different timepoints in relation to the date of depression diagnosis

Characteristic	Alcohol recorded (%)	Alcohol not recorded (%)	OR (95% CI)
Sex			
Female	2791 (14.2)	16813 (85.8)	ref
Male	3221 (19.1)	13609 (80.9)	1.38 (1.29, 1.48)
Age at depression diagnosis			
18-29	1555 (13.3)	10126 (86.7)	ref
30-39	1044 (13.8)	6507 (86.2)	1.05 (0.95, 1.17)
40-49	1329 (18.5)	5863 (81.5)	1.63 (1.47, 1.8)
50-59	943 (19.7)	3854 (80.3)	1.81 (1.62, 2.02)
60-69	545 (23.1)	1816 (76.9)	2.13 (1.86, 2.44)
>=70	596 (20.9)	2256 (79.1)	1.85 (1.61, 2.11)
Ethnicity			
White	3530 (17.4)	16739 (82.6)	ref
South Asian	152 (17.8)	700 (82.2)	1.08 (0.9, 1.3)
Black	100 (16.8)	495 (83.2)	0.92 (0.74, 1.15)
Other	58 (15.6)	315 (84.5)	0.87 (0.65, 1.15)
Mixed	43 (16.5)	217 (83.5)	1.03 (0.74, 1.44)
Unknown	194 (18.7)	843 (81.3)	1.13 (0.96, 1.33)
Geographical region			
South	3623 (14.9)	20632 (85.1)	ref
North	2389 (19.6)	9790 (80.4)	1.41 (1.31, 1.52)
IMD quintile			
1 - most deprived	1123 (14.8)	6479 (85.2)	ref
2	1125 (15.2)	6283 (84.8)	1.02 (0.91, 1.14)
3	1241 (16.4)	6324 (83.6)	1.15 (1.03, 1.28)
4	1250 (17.2)	6003 (82.8)	1.26 (1.12, 1.4)
5 - least deprived	1269 (19.3)	5316 (80.7)	1.56 (1.4, 1.75)
Time (t) since GP registration			
1-2 years	587 (15.1)	3313 (84.9)	ref
2-3 years	410 (14.7)	2374 (85.3)	0.95 (0.83, 1.1)
>3 years	3015 (16.9)	24735 (83.1)	1.11 (1, 1.23)
BMI category			
Normal (18.5 - <25.0 kg/m2)	2037 (16.6)	10217 (83.4)	ref
Underweight (<18.5kg/m2)	176 (16)	922 (84)	0.95 (0.77, 1.17)
Overweight (>=25.0 kg/m2)	3221 (18.3)	14428 (81.7)	0.98 (0.91, 1.06)
Smoking status			
Non-smoker	2199 (14.4)	13114 (85.6)	ref
Current-smoker	1911 (18.6)	8342 (81.4)	1.34 (1.23, 1.46)
Ex-smoker	1888 (17.8)	8704 (82.2)	1.14 (1.05, 1.24)
Diabetes			
None	5402 (15.6)	29219 (84.4)	ref
Type 1	63 (31.2)	139 (68.8)	2.22 (1.53, 3.22)
Type 2	452 (33.9)	883 (66.1)	1.95 (1.67, 2.27)
Type unspecified	95 (34.4)	181 (65.6)	2.18 (1.59, 2.98)
Hypertension			
None	4852 (15.3)	26916 (84.7)	ref
Present	1160 (24.9)	3506 (75.1)	1.52 (1.37, 1.7)
Liver disease			
None	5908 (16.4)	30046 (83.6)	ref
Present	104 (21.7)	376 (78.3)	0.99 (0.75, 1.31)
Anxiety			
None	4681 (16.6)	23603 (83.4)	ref
Present	1331 (16.3)	6819 (83.7)	0.98 (0.9, 1.07)
Substance abuse			
None	5951 (16.5)	23603 (83.5)	ref
Present	61 (21.2)	6819 (78.8)	0.99 (0.69, 1.44)
Depression management with antidepressants			
None	744 (17.8)	3448 (82.2)	ref
TCAs	139 (18.9)	596 (81.1)	1.13 (0.89, 1.44)
SSRIs	4771 (16.1)	24945 (83.9)	0.92 (0.82, 1.02)
Other	358 (20)	1433 (80)	1.05 (0.88, 1.25)
Depression management with talking therapies			
None	5491 (16.4)	28045 (83.6)	ref
Present	521 (18)	2377 (82)	1.2 (1.06, 1.36)

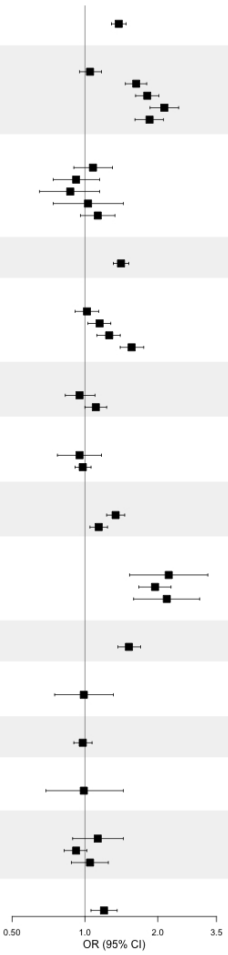

Individual characteristics of alcohol use recording in the three months before or after depression diagnosis

Supplementary material

Figure S1: Cohort selection flowchart

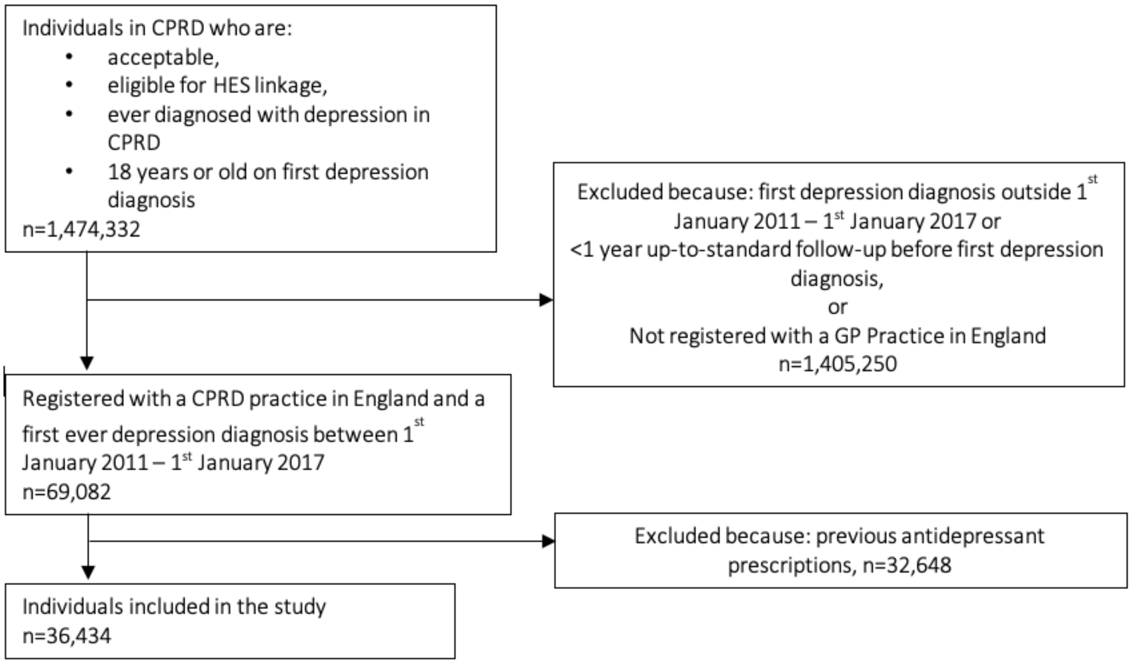

Abbreviations: CPRD – Clinical Practice Research Datalink; HES – Hospital Episode Statistics

view only

For peer review only - http://bmjopen.bmj.com/site/about/guidelines.xhtml

Algorithms used to identify BMI and smoking status

Both smoking status and BMI were identified using an algorithm that utilised primary care records to identify the status recorded closest to the date of depression diagnosis. For BMI, Read codes for BMI category were not used because they are rarely recorded. Instead, BMI was calculated using height and weight measures recorded closest to the date of depression diagnosis. In the algorithm, records within -1 year to +1 month of the date of depression diagnosis were regarded as the best, +1 month to +1 year from the date of depression diagnosis as second best, the nearest prior to the year before the date of depression diagnosis as the third best, and within +1 year from the date of depression diagnosis as the worst. BMI was categorised as underweight (<18.5kg/m^2), normal (18.5 – <25.0 kg/m^2) or overweight (≥25.0 kg/m^2). Smoking status was classified as current smoker, ex-smoker, or non-smoker.

For peer review only

Table 1: Characteristics of individuals in the study and the odds of alcohol-use recording in the three months before or after depression diagnosis (data are N(%) unless otherwise specified)

	Alcohol use recording in primary care electronic health records		Age, sex, ethnicity, and deprivation adjusted odds ratio for alcohol use recording (95% CI)[b]
	Recorded [a]	Not recorded	
	N=6,012 (16.5%)	N=30,422 (83.5%)	
Sex			
Female	2,791 (14.2)	16,813 (85.8)	reference
Male	3,221 (19.1)	13,609 (80.9)	1.38 (1.29, 1.48)
Age at depression diagnosis (years)			
18-29	1,555 (13.3)	10,126 (86.7)	reference
30-39	1,044 (13.8)	6,507 (86.2)	1.05 (0.95, 1.17)
40-49	1,329 (18.5)	5,863 (81.5)	1.63 (1.47, 1.80)
50-59	943 (19.7)	3,854 (80.3)	1.81 (1.62, 2.02)
60-69	545 (23.1)	1,816 (76.9)	2.13 (1.86, 2.44)
≥70	596 (20.9)	2,256 (79.1)	1.85 (1.61, 2.11)
Ethnicity			
White	3,530 (17.4)	16,739 (82.6)	reference
South Asian	152 (17.8)	700 (82.2)	1.08 (0.90, 1.30)
Black	100 (16.8)	495 (83.2)	0.92 (0.74, 1.15)
Other	58 (15.6)	315 (84.5)	0.87 (0.65, 1.15)
Mixed	43 (16.5)	217 (83.5)	1.03 (0.74, 1.44)
Unknown	194 (18.7)	843 (81.3)	1.13 (0.96, 1.33)
Missing	*1,935 (14.8)*	*11,113 (85.17)*	
Geographical region			
South	3,623 (14.9)	20,632 (85.1)	reference
North	2,389 (19.6)	9,790 (80.4)	1.41 (1.31, 1.52)
IMD quintile			
1 – most deprived	1,123 (14.8)	6,479 (85.2)	reference
2	1,125 (15.2)	6,283 (84.8)	1.02 (0.91, 1.14)
3	1,241 (16.4)	6,324 (83.6)	1.15 (1.03, 1.28)
4	1,250 (17.2)	6,003 (82.8)	1.26 (1.12, 1.40)
5 – least deprived	1,269 (19.3)	5,316 (80.7)	1.56 (1.40, 1.75)
Missing	*4 (19.1)*	*17 (80.9)*	
Time (t) since GP registration (years)			
1<t≤2	587 (15.1)	3,313 (84.9)	reference
2<t≤3	410 (14.7)	2,374 (85.3)	0.95 (0.83, 1.10)
>3	3,015 (16.9)	24,735 (83.1)	1.11 (1.00, 1.23)
BMI Category			
Normal (18.5 – <25.0 kg/m^2)	2,037 (16.6)	10,217 (83.4)	reference
Underweight (<18.5kg/m^2)	176 (16.0)	922 (84.0)	0.95 (0.77, 1.17)
Overweight (≥25.0 kg/m^2)	3,221 (18.3)	14,428 (81.7)	0.98 (0.91, 1.06)
Missing	*578 (10.6)*	*4,855 (89.4)*	
Smoking Status			
Non-smoker	2,199 (14.4)	13,114 (85.6)	reference
Current smoker	1,911 (18.6)	8,342 (81.4)	1.34 (1.23, 1.46)
Ex-smoker	1,888 (17.8)	8,704 (82.2)	1.14 (1.05, 1.24)

For peer review only

Missing	*14 (5.1)*	*262 (94.9)*	
Comorbidities [c]			
Diabetes mellitus			
None	5,402 (15.6)	29,219 (84.4)	reference
Type 1	63 (31.2)	139 (68.8)	2.22 (1.53, 3.22)
Type 2	452 (33.9)	883 (66.1)	1.95 (1.67, 2.27)
Type unspecified	95 (34.4)	181 (65.6)	2.18 (1.59, 2.98)
Hypertension			
None	4,852 (15.3)	26,916 (84.7)	reference
Present	1,160 (24.9)	3,506 (75.1)	1.52 (1.37, 1.70)
Liver Disease			
None	5,908 (16.4)	30,046 (83.6)	reference
Yes	104 (21.7)	376 (78.3)	0.99 (0.75, 1.31)
Anxiety			
None	4,681 (16.6)	23,603 (83.4)	reference
Yes	1,331 (16.3)	6,819 (83.7)	0.98 (0.90, 1.07)
Substance Abuse			
None	5,951 (16.5)	30,195 (83.5)	reference
Yes	61 (21.2)	227 (78.8)	0.99 (0.69, 1.44)
Depression Management			
Antidepressant class			
None	744 (17.8)	3,448 (82.2)	reference
TCAs	139 (18.9)	596 (81.1)	1.13 (0.89, 1.44)
SSRIs	4,771 (16.1)	24,945 (83.9)	0.92 (0.82, 1.02)
Other	358 (20.0)	1,433 (80.0)	1.05 (0.88, 1.25)
Talking Therapies			
No	5,491 (16.4)	28,045 (83.6)	reference
Yes	521 (18.0)	2,377 (82.0)	1.20 (1.06, 1.36)

Abbreviations: TCAs – tricyclic antidepressants; SSRIs – selective serotonin uptake inhibitors
[a] The first instance of alcohol use recording in the three months before or after depression diagnosis
[b] Odds ratio adjusted for sociodemographic confounders (age, sex, ethnicity and deprivation)

For peer review only

The RECORD statement – checklist of items, extended from the STROBE statement, that should be reported in observational studies using routinely collected health data.

	Item No.	STROBE items	Location in manuscript where items are reported	RECORD items	Location in manuscript where items are reported
**Title and abstract**					
	1	(a) Indicate the study's design with a commonly used term in the title or the abstract (b) Provide in the abstract an informative and balanced summary of what was done and what was found	1-22	RECORD 1.1: The type of data used should be specified in the title or abstract. When possible, the name of the databases used should be included.<br><br>RECORD 1.2: If applicable, the geographic region and timeframe within which the study took place should be reported in the title or abstract.<br><br>RECORD 1.3: If linkage between databases was conducted for the study, this should be clearly stated in the title or abstract.	1-2
**Introduction**					
Background rationale	2	Explain the scientific background and rationale for the investigation being reported	4		
Objectives	3	State specific objectives, including any prespecified hypotheses	4		
**Methods**					
Study Design	4	Present key elements of study design early in the paper	5		
Setting	5	Describe the setting, locations, and relevant dates, including periods of recruitment, exposure, follow-up, and data collection	5		

For peer review only

Participants	6	*(a) Cohort study* - Give the eligibility criteria, and the sources and methods of selection of participants. Describe methods of follow-up<br>*Case-control study* - Give the eligibility criteria, and the sources and methods of case ascertainment and control selection. Give the rationale for the choice of cases and controls<br>*Cross-sectional study* - Give the eligibility criteria, and the sources and methods of selection of participants<br><br>*(b) Cohort study* - For matched studies, give matching criteria and number of exposed and unexposed<br>*Case-control study* - For matched studies, give matching criteria and the number of controls per case	5	RECORD 6.1: The methods of study population selection (such as codes or algorithms used to identify subjects) should be listed in detail. If this is not possible, an explanation should be provided.<br><br>RECORD 6.2: Any validation studies of the codes or algorithms used to select the population should be referenced. If validation was conducted for this study and not published elsewhere, detailed methods and results should be provided.<br><br>RECORD 6.3: If the study involved linkage of databases, consider use of a flow diagram or other graphical display to demonstrate the data linkage process, including the number of individuals with linked data at each stage.	5-6 and supplementary material
Variables	7	Clearly define all outcomes, exposures, predictors, potential confounders, and effect modifiers. Give diagnostic criteria, if applicable.	5-6 and supplementary material	RECORD 7.1: A complete list of codes and algorithms used to classify exposures, outcomes, confounders, and effect modifiers should be provided. If these cannot be reported, an explanation should be provided.	5
Data sources/ measurement	8	For each variable of interest, give sources of data and details of methods of assessment (measurement).<br>Describe comparability of assessment methods if there is more than one group	5-6 and supplementary material		

For peer review only

Bias	9	Describe any efforts to address potential sources of bias	5-6		
Study size	10	Explain how the study size was arrived at	5-6		
Quantitative variables	11	Explain how quantitative variables were handled in the analyses. If applicable, describe which groupings were chosen, and why	5-6		
Statistical methods	12	(a) Describe all statistical methods, including those used to control for confounding<br>(b) Describe any methods used to examine subgroups and interactions<br>(c) Explain how missing data were addressed<br>(d) *Cohort study* - If applicable, explain how loss to follow-up was addressed<br>*Case-control study* - If applicable, explain how matching of cases and controls was addressed<br>*Cross-sectional study* - If applicable, describe analytical methods taking account of sampling strategy<br>(e) Describe any sensitivity analyses	6		
Data access and cleaning methods		..		RECORD 12.1: Authors should describe the extent to which the investigators had access to the database population used to create the study population.	5

For peer review only

				RECORD 12.2: Authors should provide information on the data cleaning methods used in the study.	
Linkage		..		RECORD 12.3: State whether the study included person-level, institutional-level, or other data linkage across two or more databases. The methods of linkage and methods of linkage quality evaluation should be provided.	5
**Results**					
Participants	13	(a) Report the numbers of individuals at each stage of the study (*e.g.*, numbers potentially eligible, examined for eligibility, confirmed eligible, included in the study, completing follow-up, and analysed)<br>(b) Give reasons for non-participation at each stage.<br>(c) Consider use of a flow diagram	Supplementary material	RECORD 13.1: Describe in detail the selection of the persons included in the study (*i.e.,* study population selection) including filtering based on data quality, data availability and linkage. The selection of included persons can be described in the text and/or by means of the study flow diagram.	Supplementary material
Descriptive data	14	(a) Give characteristics of study participants (*e.g.*, demographic, clinical, social) and information on exposures and potential confounders<br>(b) Indicate the number of participants with missing data for each variable of interest<br>(c) *Cohort study* - summarise follow-up time (*e.g.*, average and total amount)	7, 11-12		
Outcome data	15	*Cohort study* - Report numbers of outcome events or summary measures over time<br>*Case-control study* - Report numbers in each exposure	8-10		

For peer review only

		category, or summary measures of exposure<br>*Cross-sectional study* - Report numbers of outcome events or summary measures			
Main results	16	(a) Give unadjusted estimates and, if applicable, confounder-adjusted estimates and their precision (e.g., 95% confidence interval). Make clear which confounders were adjusted for and why they were included<br>(b) Report category boundaries when continuous variables were categorized<br>(c) If relevant, consider translating estimates of relative risk into absolute risk for a meaningful time period	11-12		
Other analyses	17	Report other analyses done—e.g., analyses of subgroups and interactions, and sensitivity analyses	N/A		
**Discussion**					
Key results	18	Summarise key results with reference to study objectives	13		
Limitations	19	Discuss limitations of the study, taking into account sources of potential bias or imprecision. Discuss both direction and magnitude of any potential bias	14	RECORD 19.1: Discuss the implications of using data that were not created or collected to answer the specific research question(s). Include discussion of misclassification bias, unmeasured confounding, missing data, and changing eligibility over time, as they pertain to the study being reported.	14-15
Interpretation	20	Give a cautious overall interpretation of results considering objectives,	15		

For peer review only

		limitations, multiplicity of analyses, results from similar studies, and other relevant evidence			
Generalisability	21	Discuss the generalisability (external validity) of the study results	15		
**Other Information**					
Funding	22	Give the source of funding and the role of the funders for the present study and, if applicable, for the original study on which the present article is based	17		
Accessibility of protocol, raw data, and programming code		..		RECORD 22.1: Authors should provide information on how to access any supplemental information such as the study protocol, raw data, or programming code.	17

*Reference: Benchimol EI, Smeeth L, Guttmann A, Harron K, Moher D, Petersen I, Sørensen HT, von Elm E, Langan SM, the RECORD Working Committee. The REporting of studies Conducted using Observational Routinely-collected health Data (RECORD) Statement. *PLoS Medicine* 2015; in press.

*Checklist is protected under Creative Commons Attribution (CC BY) license.

For peer review only
